# Impact of prophylactic vaccination strategies on Ebola virus transmission: A modeling analysis

**DOI:** 10.1371/journal.pone.0230406

**Published:** 2020-04-27

**Authors:** Ravi Potluri, Amit Kumar, Vikalp Maheshwari, Charlie Smith, Valerie Oriol Mathieu, Kerstin Luhn, Benoit Callendret, Hitesh Bhandari

**Affiliations:** 1 SmartAnalyst Inc., New York, New York, United States of America; 2 SmartAnalyst India Pvt. Ltd., Gurgaon, India; 3 Janssen Vaccines & Prevention, Raritan, New Jersey, United States of America; 4 Janssen Vaccines & Prevention B.V., Leiden, The Netherlands; Stanford University School of Medicine, UNITED STATES

## Abstract

Ebola epidemics constitute serious public health emergencies. Multiple vaccines are under development to prevent these epidemics and avoid the associated morbidity and mortality. Assessing the potential impact of these vaccines on morbidity and mortality of Ebola is essential for devising prevention strategies. A mean-field compartmental stochastic model was developed for this purpose and validated by simulating the 2014 Sierra Leone epidemic. We assessed the impacts of prophylactic vaccination of healthcare workers (HCW) both alone and in combination with the vaccination of the general population (entire susceptible population other than HCW). The model simulated 8,706 (95% confidence intervals [CI]: 478–21,942) cases and 3,575 (95%CI: 179–9,031) deaths in Sierra Leone, in line with WHO-reported statistics for the 2014 epidemic (8,704 cases and 3,587 deaths). Relative to this base case, the model then estimated that prophylactic vaccination of only 10% of HCW will avert 12% (95% CI: 6%-14%) of overall cases and deaths, while vaccination of 30% of HCW will avert 34% of overall cases (95% CI: 30%-64%) and deaths (95% CI: 30%-65%). Prophylactic vaccination of 1% and 5% of the general population in addition to vaccinating 30% of HCW was estimated to result in reduction in cases by 44% (95% CI: 39%-61%) and 72% (95% CI: 68%-84%) respectively, and deaths by 45% (95% CI: 40%-61%) and 74% (95% CI: 70%-85%) respectively. Prophylactic vaccination of even small proportions of HCW is estimated to significantly reduce incidence of Ebola and associated mortality. The effect is greatly enhanced by the additional vaccination even of small percentages of the general population. These findings could be used to inform the planning of prevention strategies.

## Introduction

Ebola hemorrhagic fever, caused by the Ebola viruses, represents a significant public health problem given its severity, high case-fatality rates, and repeated occurrence, with more than 25 outbreaks and epidemics having been reported since 1976 [1]. Outbreaks and epidemics are on the rise, with more cases and deaths having been reported in the past 10 years than at any point in Ebola history, including the epidemic in West Africa that was officially declared a “public health emergency of international concern” by the World Health Organization (WHO) in 2014 [2–4]. By the end of this emergency in March 2016, a total of 28,610 cases and 11,308 deaths had been reported [5]. While the factors that influenced the rapid spread of the infection and the eventual devastation that resulted are complex, enhanced intermixing of populations facilitated by efficient transportation links between rural villages and densely-populated urban areas [3] may have played a role. Once the infection was widespread, factors that were implicated in the inability to rapidly control the epidemic included traditional burial practices, weak healthcare infrastructure, sub-optimally trained healthcare workers (HCW), and non-cooperation by families [6,7]. Moreover, no vaccine or treatment was available during the initial stages of the epidemic, and it was only during the later stages that an investigational vaccine was administered in Guinea and Sierra Leone using a ring vaccination strategy [8,9].

Multiple Ebola vaccine candidates are currently in development [10]. While prophylactic Ebola vaccination is not part of the standard Ebola virus disease prevention strategy yet, recent regulatory actions are being considered to be major steps towards greater availability of Ebola vaccines in near future in high-risk countries [11]. These actions include the European Commission’s decision to grant a conditional marketing authorization to a vaccine candidate in November 2019 (which was followed by WHO prequalification), its subsequent independent approval by the United States Food and Drug Administration, and submission of a prophylactic vaccine candidate for accelerated assessment by Committee for Medicinal Products for Human Use of the European Medicines Agency. Given these recent developments, multiple relevant international public health institutions and governments of affected countries are considering stockpiling of vaccines both for emergency use in the event of an outbreak or epidemic and/or for preventive vaccination of selective at-risk populations (including HCW in endemic areas who carry a significantly higher risk of infection) and residents of densely-populated urban areas [12]. Henao-Restrepo and colleagues have postulated that effective ring vaccination can help control Ebola outbreaks [9], while Walldorf and colleagues have stated that rapid vaccination of vulnerable populations can constitute an integral part of the emergency response strategy [12]. However, widespread vaccination in the midst of an outbreak or epidemic can pose challenges including estimation and allocation of resources to ensure timely vaccine availability, acceptance by the population, and speedy deployment over large geographical areas [12]. The persistent epidemic in the Democratic Republic of Congo that has been ongoing since August 2018, with approximately 3,428 cases and 2,246 deaths having been reported as of February 2020 [13], has put large cities and neighboring countries at risk and reinforces the urgent need to formulate and implement effective prophylactic vaccination strategies [14].

In light of the high public health importance of Ebola hemorrhagic fever, it is necessary to evaluate the impact of different prophylactic vaccination strategies, whether they involve entire populations or sub-populations selected on the basis of risk of infection.

Multiple mathematical modeling analyses related to Ebola hemorrhagic fever have been undertaken to forecast peak incidence and size of outbreaks [15]. They have evaluated shifts in disease transmission dynamics during epidemics [16], identified factors contributing to the recurrence and persistence of outbreaks [17], assessed the population-level impact of quarantine on disease transmission dynamics [18], estimated size and duration of outbreaks with and without vaccine use [19], assessed the role of sexual transmission in spread of infection during outbreaks [20], captured real-time disease dynamics in the midst of outbreaks [21], projected the short- and long-term course of outbreaks [22], evaluated the effectiveness of control measures (e.g., isolation of cases, safe burials, and social distancing) implemented to stop outbreaks [23], quantified the impact of vaccination on the spatiotemporal dynamics of disease transmission [24], evaluated vaccination of HCW [25], and evaluated the impact of vaccination on the course of Ebola epidemics [23,25–27].

This study aimed to evaluate, with the help of an appropriate mathematical model, the impact of prophylactic vaccination of varying proportions of HCW and the general population (the entire susceptible population other than HCW) on the size of a potential Ebola outbreak.

## Methods

### Overview

A central element of the study was the development of a mathematical model that could simulate an outbreak and facilitate analysis of different vaccination scenarios, from “no vaccination” to vaccination of different proportions of HCW and the general population. The model accommodated the following variables: i) timing of vaccination in relation to the outbreak, whether prior to (proactive) or following (reactive), ii) extent and rate of vaccination, and iii) vaccine characteristics, including onset of protection and duration of protection. Given that the principal objective of the study is to evaluate the impact of prophylactic vaccination of varying proportions of HCW and the general population on the size of a potential Ebola outbreak, model variables related to prophylactic vaccination have been explained here and those specific to reactive vaccination and/or vaccine characteristics have been explained in the supporting material. Findings from analysis of a reactive vaccination strategy were only used to compare with the results of the analysis of prophylactic vaccination strategies.

Creation of the model involved a three-step approach: 1) selection of an appropriate model construct that could replicate past outbreaks/epidemics, 2) calibration of the model to reflect a real-life epidemic (we chose the 2014 Sierra Leone epidemic for the size of the epidemic and the availability of consistent weekly reports on cases and deaths), and 3) adaptation of the model to accommodate vaccination of the susceptible population. These involved a review of the literature to identify an appropriate modelling approach, design of the model structure, and identification of data related to the 2014 Sierra Leone epidemic. This was followed by a) fitting of geography- and disease-related parameters by deterministically aligning the model with this epidemic, b) creation of a stochastic model to run simulations, c) validation of the stochastic model through comparison of model mean outcomes with confirmed cases and deaths reported for the Sierra Leone epidemic of 2014 by the WHO [28] and Fang et al. [29], and d) adaptation of the validated model to incorporate key aspects of vaccination.

### Choice of model and design of the model structure

**Fig 1:**
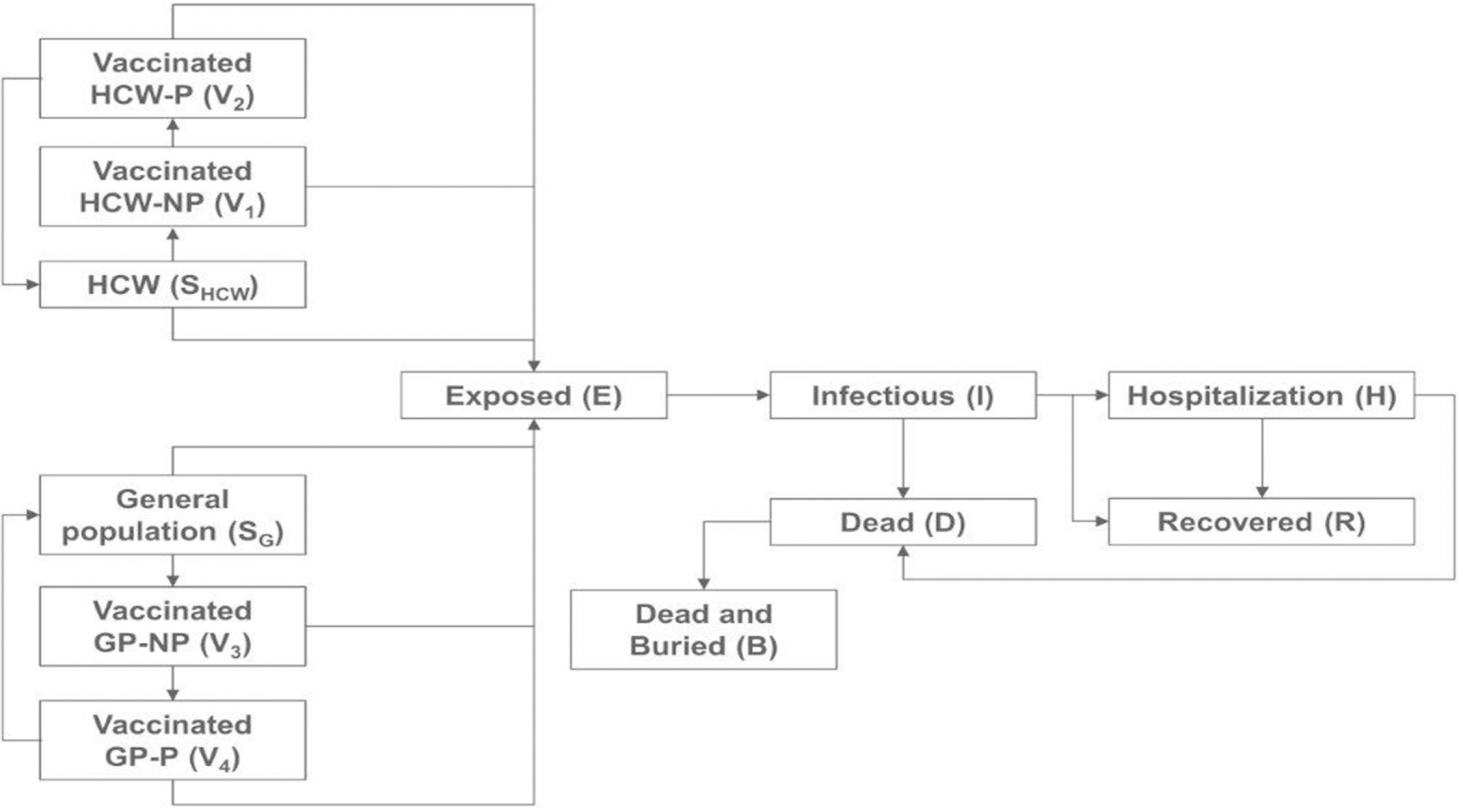
Expanded SEIR framework-based model differentiating between HCW and the general population, permitting movement between populations based on infection status, and accommodating vaccination. Abbreviations: S, susceptible population; GP, general population; HCW, healthcare workers; General population, total population minus HCW; NP, not protected; P, protected.

A literature search was carried out to identify the type of model which was best suited to replicate a past outbreak. Results of the literature search and the decision to use the stochastic mean-field compartmental model are detailed in the S1 File. Mean-field compartmental models are based on the ‘susceptible, exposed, infected, and removed (SEIR)’ framework or its variants, with the population being divided into and moving between multiple states of infection over time and each state being assumed to comprise a homogenous population. However, the generalized SEIR framework-based model neither explicitly differentiates between HCW and the general population nor provides for vaccination, both aspects essential to address the objectives of the study. The standard generalized model was therefore customized for this study by expanding it to facilitate differentiation between HCW and the general population and to permit movement between susceptible, exposed, and infected populations while also accounting for both prophylactic and reactive vaccination (Fig 1).

### Model parameterization and validation

**Fig 2:**
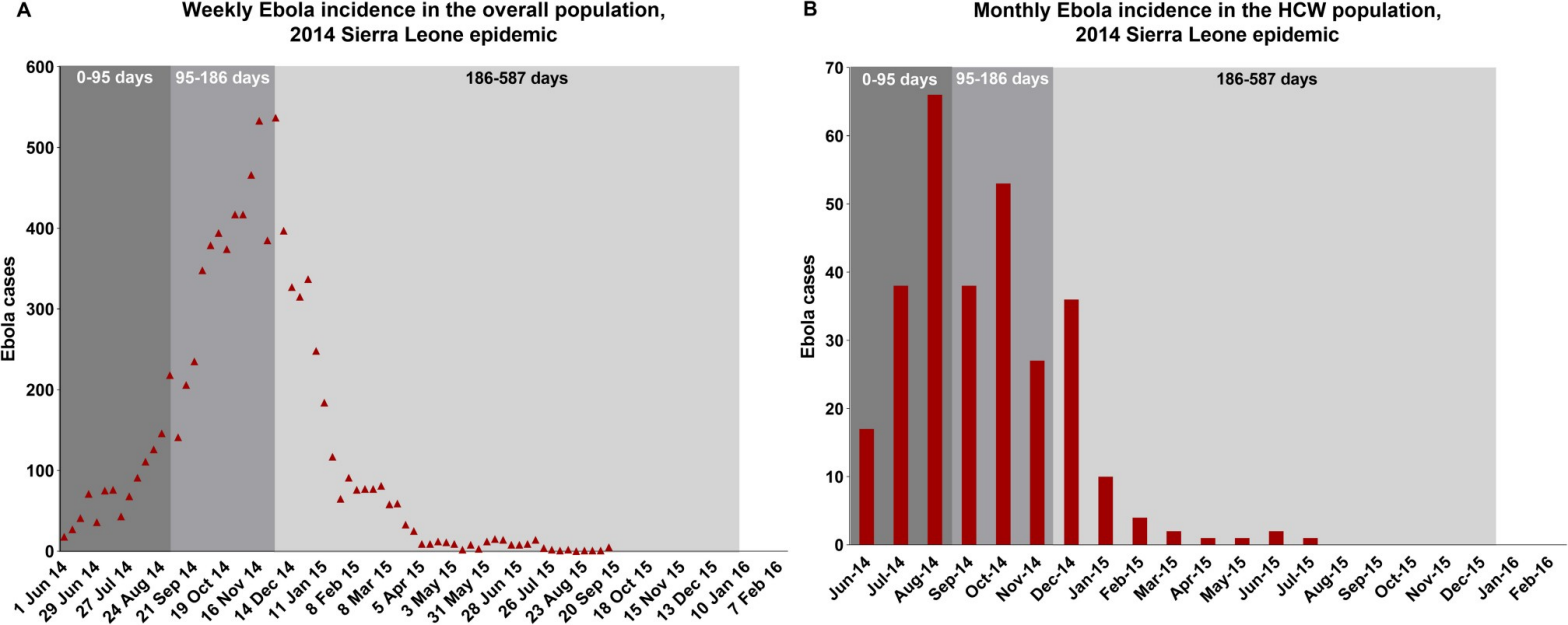
Reported Ebola cases for the 2014 Sierra Leone epidemic–among overall population (weekly cases) and in HCW (monthly cases). Abbreviation: HCW, Healthcare workers; WHO, World Health Organization. Data for the figures were obtained from the WHO [31] and the study by Fang and colleagues [29].

The 2014 epidemic in Sierra Leone was simulated with the help of disease-specific parameters that were derived from a literature search and geographic/epidemic-specific parameters that were estimated by fitting the model to minimize simultaneously both the squared differences between weekly cases in the overall population as modeled and as reported by WHO for the 2014 epidemic and the squared differences among the HCW between monthly cases as modeled and as reported for the 2014 epidemic [28,29]. For this exercise, the entirety of the model horizon (May 28, 2014–December 31, 2015) was divided into three periods for fitting: a) an initial 95-day period that saw a sharp increase in Ebola cases among HCW and relatively slower increase in cases among the general population; b) the next period between days 95 and 186 that witnessed a decline in cases among HCW but an increase in cases among the general population; and c) a final period between days 186 and 587 characterized by a decline in cases among both HCW and the general population (Fig 2). The parameters being fitted were separately estimated for these three periods. The model parameters so derived from the fitting process are presented in Table 1. Using these parameters, the model was stochastically simulated by applying the direct method algorithm of Gillespie [30]. The cumulative cases and deaths from the model, averaged across 5000 simulations, were compared with WHO data relating to the 2014 Sierra Leone epidemic to check for closeness of fit.

**Table 1:** Model fitting using individual and disease/epidemic-specific parameters.

Parameter	Description		0–95 Days	95–186 Days	186–587 Days	Source
		**Disease/Epidemic-specific parameters**				
N	Size of the total population–Sierra Leone (2014)		7,017,144			World Bank Group [32]
S_HCW_	HCW		1153			Evans et al. [33]
1/σ	Mean latency period		7 days	7 days	7 days	Legrand et al. [34]
1/γ_D_	Mean duration from death to burial		2 days	2 days	2 days	Legrand et al. [34]
1/α	Mean duration from onset of infection to hospitalization		2.4 days	2.4 days	2.2 days	Fitted
β_I→HCW_	Transmission rate from infectious individuals to HCW (In days−1)		117.8	15.0	5.1	Fitted
β_H→HCW_	Transmission rate from hospitalized individuals to HCW (In days−1)		189.21	23.64	8.88	Fitted
β_D→HCW_	Transmission rate from dead but not buried individuals to HCW (In days−1)		0.0726	0.0511	0.0450	Fitted
β_I→NHCW_	Transmission rate from infectious individuals to the non-HCW/general population (In days−1)		0.635	0.594	0.425	Fitted
β_H→NHCW_	Transmission rate from hospitalized individuals to the non-HCW/general population (In days−1)		0.0020	0.0010	0.0005	Fitted
β_D→NHCW_	Transmission rate from dead but not buried individuals to the non-HCW/general population (In days−1)		0.0726	0.0511	0.0450	Fitted
δ_1_	Case-fatality rate among non-hospitalized infectious individuals		0.46	0.21	0.68	Fitted
δ_2_	Case-fatality rate among hospitalized individuals		0.46	0.21	0.68	Fitted
1/γ	Mean duration from onset of infection to death/recovery		6 days	6 days	6 days	Fitted
1/γ_H_	Mean duration from hospitalization to death/recovery		6.2 days	8.3 days	16 days	Fitted
K_1_	Proportion of HCW in the total population at the start of the epidemic		0.016%			Calculated

Abbreviations: HCW, healthcare workers; Non-HCW–overall population minus healthcare workers; WHO, World Health Organization

### Basic reproduction number

A key parameter which can help understand the spread of an infection is the basic reproduction number (R_0_), defined as the number of secondary cases generated by an infected case over the course of his/her infectious period in the absence of any control measures. If R_0_>1, the outbreak is expected to spread, while if R_0_<1, the outbreak is likely to subside. We used the next generation matrix approach described by Diekmann and colleagues [35] to calculate the basic reproduction number (R_0_). While the detailed derivation of R_0_ is available in the S1 File, the following equation was used to calculate this key parameter:

$$R_{0}=\left[\frac{K_{1}\beta_{I\to HCW}+(1-K_{1})\beta_{I\to NHCW}}{\left(\alpha +\gamma \right)}+\alpha (\frac{K_{1}\beta_{H\to HCW}+(1-K_{1})\beta_{H\to NHCW}}{\gamma_{H}(\alpha +\gamma )})+\frac{(\delta_{2}\alpha +\delta_{1}\gamma )\beta_{D}}{\gamma_{D}(\alpha +\gamma )}\right]$$

### Scenarios analyzed

The model, validated as described above, was employed for the following evaluations: i) impact of prophylactic vaccination of a) 10% and b) 30% of all HCW compared with a no-vaccination scenario; and ii) impact of prophylactic vaccination of a) 1% and b) 5% of the general population in addition to vaccination of 30% of the HCW, compared both with a no-vaccination scenario and vaccination of 30% of HCW alone. To carry out these evaluations, the model was run to simulate the number of cases and deaths in each scenario, and differences in such cases and deaths between any two scenarios being compared were computed. The proportions of populations to be vaccinated considered for these evaluations were chosen to be at the lower end of the scale in order to evaluate the impact of even modest levels of vaccination. It was assumed in this exercise that vaccinated individuals in each scenario were fully protected, based on a theoretical vaccine efficacy of 100%, both at the time of and through the course of the epidemic.

For evaluation of these scenarios, the values of the key model parameters were all based on the 2014 Sierra Leone epidemic. The vaccination strategy related parameters used in the model are listed and described in S4 Table along with all other model parameters. We also carried out a sensitivity analysis using different values for a subset of these fitted parameters (S5 Table) in order to evaluate consistency in the impact of prophylactic vaccination.

## Results

### Model parameterization and validation

**Fig 3:**
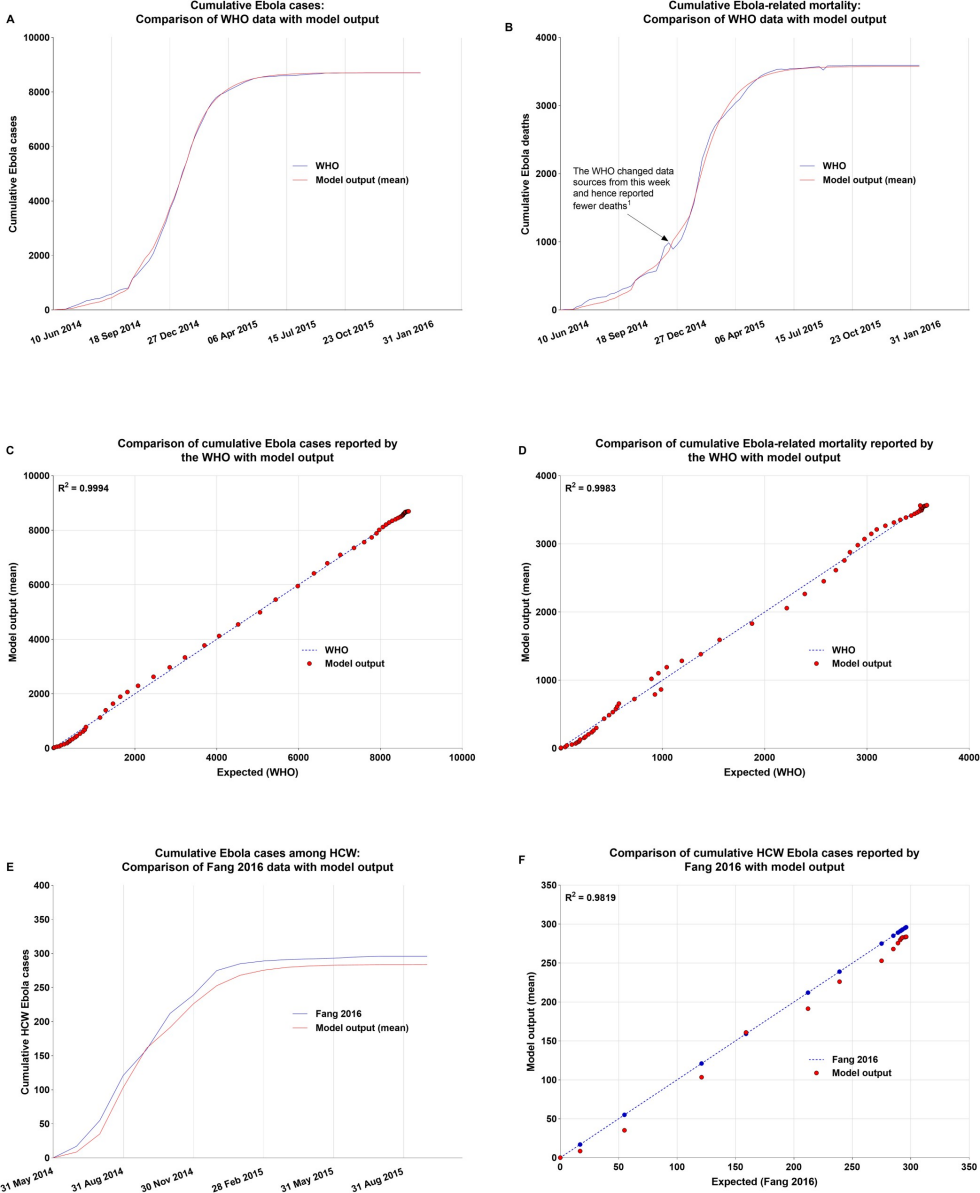
Comparison of the output of the base case model (without vaccination) with published data. A, Cumulative Ebola cases. B, Cumulative Ebola mortality. C, Comparison of cumulative Ebola cases reported by the WHO with model output. D, Comparison of cumulative Ebola-related mortality reported by the WHO with model output. E, Cumulative Ebola cases among HCW. F, Comparison of cumulative HCW Ebola cases reported by Fang and colleagues with model output. Abbreviation: HCW, Healthcare Workers; WHO, World Health Organization.

The parameters fitted to the weekly (general population)/monthly (HCW) cases reported for the 2014 epidemic in Sierra Leone separately for the three periods of the epidemic can be seen in Table 1. Comparison of the output of the stochastic simulation of the fitted model with published data [31] confirmed a good fit (Fig 3A–3F). The model simulated 8,706 cases and 3,575 deaths, compared to 8,704 cases and 3,587 deaths reported by WHO. Of the total number of Ebola cases in the model, 284 cases were estimated to have occurred among the HCW, which was similar to the number (296) reported by Fang and colleagues [29]. R_0_ was calculated to be 1.34. This is consistent with previously published estimates of R_0_ for the 2014 Sierra Leone epidemic (1.26 < R_0_ < 2.53) [26].

### Impact of vaccination

**Fig 4:**
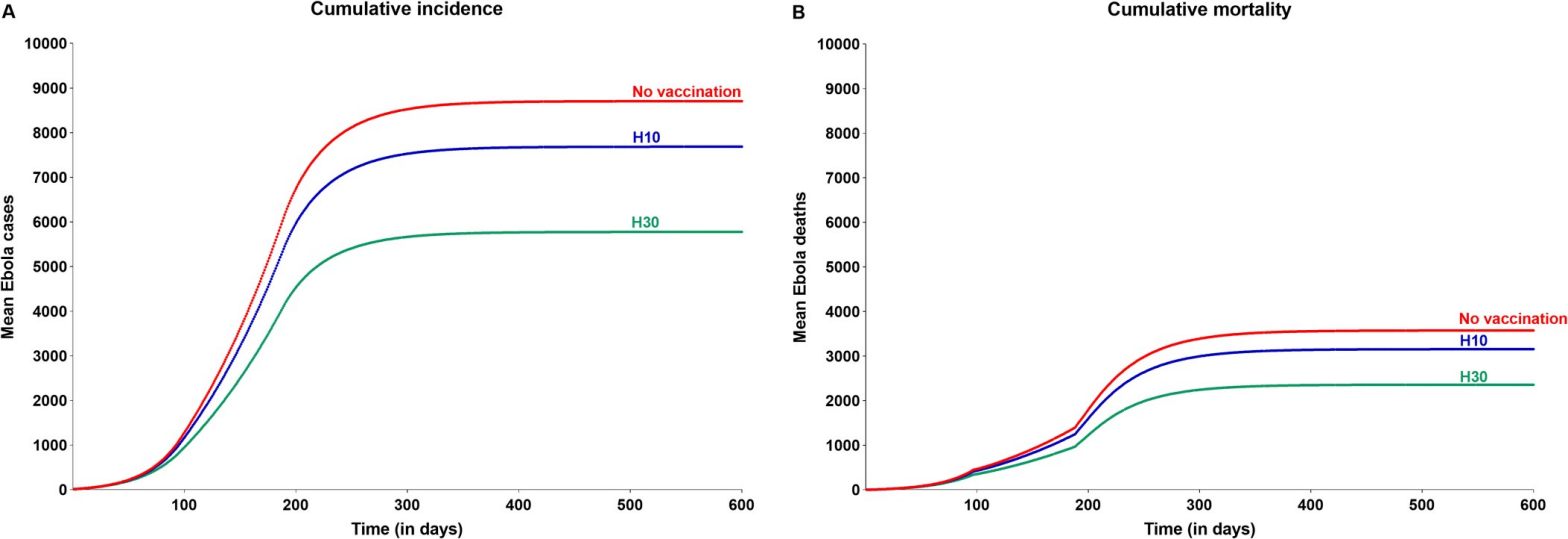
Impact of prophylactic vaccination of different proportions of HCW on cumulative incidence and mortality associated with Ebola virus disease. Abbreviations: HCW, health care worker; H10, vaccination of 10% of all HCW; H30, vaccination of 30% of all HCW.

In the evaluation comparing the size of the epidemic in the event of prophylactic vaccination of different proportions of HCW with that of the above base case when no preventive vaccination was carried out, it was determined that prophylactic vaccination of as few as 10% of all HCW (115, who represent just 0.0016% of the overall Sierra Leone population) will help reduce both cases and deaths at the overall population level by 12% (1,021 cases and 420 deaths averted). The corresponding reduction with prophylactic vaccination of 30% of all HCW (345) was 34% (2,930 cases and 1,217 deaths averted) (Fig 4 and Table 2).

**Table 2:** Impact of prophylactic vaccination of healthcare workers and general population on cumulative incidence and mortality associated with Ebola virus disease.

Parameter	No vaccination	10% of HCW vaccinated	30% of HCW vaccinated	30% of HCW + 1% of GP vaccinated	30% of HCW + 5% of GP vaccinated
Number vaccinated	0	115	345	70,504	351,143
Cumulative cases (IQR; 95%CI)	8,706 (4,427–12,059; 478–21,942)	7,685 (3,905–10,588; 482–19,451)	5,776 (2,687–8,164; 121–15,339)	4,888 (2,250–6,904; 183–13,371)	2,469 (1,043–3,486; 77–6,959)
Proportion of cases averted vs no vaccination (IQR; 95%CI)	–	12% (11%-12%; 6%-14%)	34% (32%-39%; 30%-64%)	44% (43%-49%; 39%-61%)	72% (71%-76%; 68%-84%)
Proportion of cases averted vs vaccination of 30% of HCW (IQR; 95%CI)	–	–	–	15% (15%-16%; (-)17%-19%)	57% (57%-61%; 37%-65%)
Cumulative deaths (IQR; 95%CI)	3,575 (1,821–4,950; 179–9,031)	3,155 (1,598–4,342; 180–7,911)	2,358 (1,082–3,338; 49–6,213)	1,966 (897–2,784; 71–5,396)	940 (391–1,331; 32–2,672)
Proportion of deaths averted vs no vaccination (IQR; 95%CI)	–	12% (11%-12%; 6%-14%)	34% (32%-40%; 30%-65%)	45% (44%-51%; 40%-61%)	74% (73%-78%; 70%-85%)
Proportion of deaths averted vs vaccination of 30% of HCW (IQR; 95%CI)	–	–	–	17% (16%-18%; (-)21%-20%)	60% (60%-63%; 36%-67%)

Abbreviations: CI, credible interval; HCW, healthcare workers; IQR, interquartile range; GP, general population

**Fig 5:**
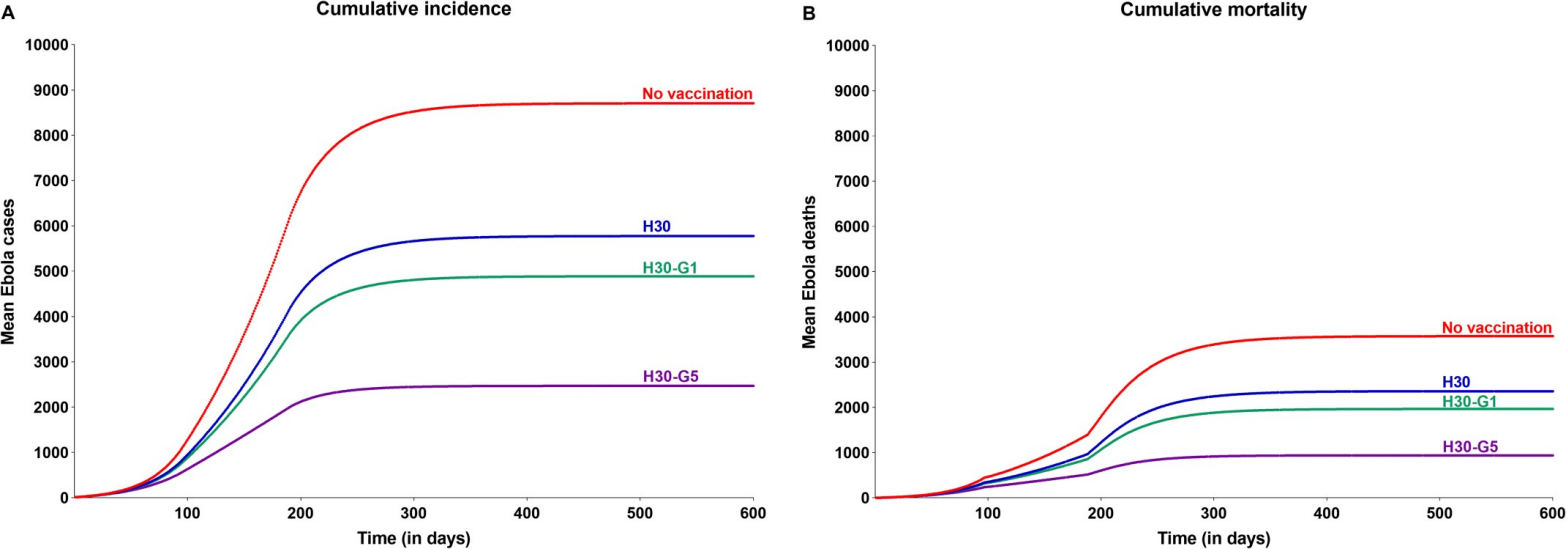
Impact of prophylactic vaccination of different proportions of the general population, in addition to vaccination of 30% of HCW, on cumulative incidence and mortality associated with Ebola virus disease. Abbreviations: G, general population; HCW, health care worker; H30, vaccination of 30% of all HCW; H30-G1, vaccination of 30% of all HCW plus 1% of the general population; H30-G5, vaccination of 30% of all HCW plus 5% of the general population.

**Fig 6:**
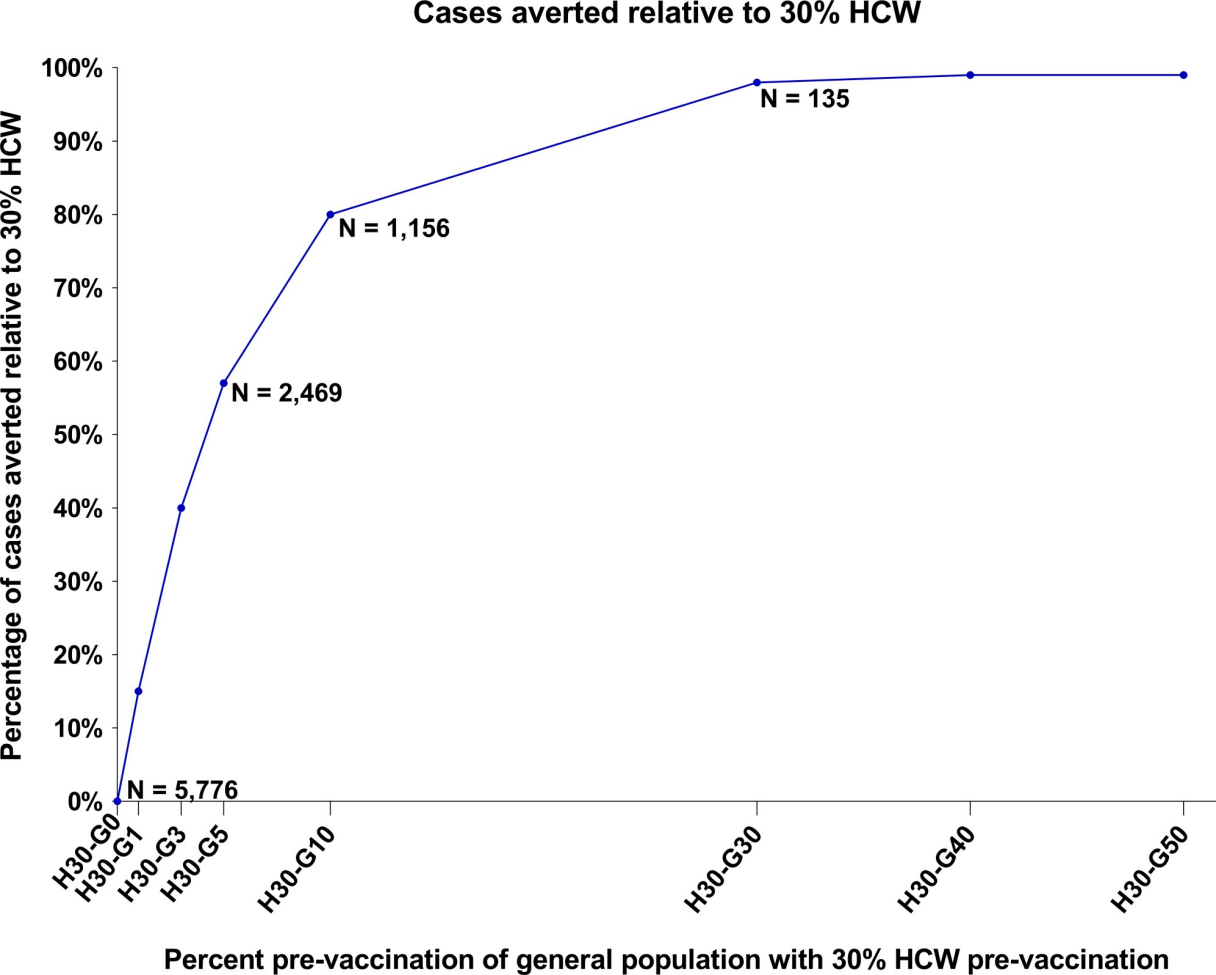
Incremental benefit of prophylactic vaccination of the general population as a function of the vaccination coverage. Abbreviations: HCW, health care worker; H30, vaccination of 30% of all HCW; H30-Gx, vaccination of 30% of all HCW plus x% of the general population.

Prophylactic vaccination of the general population was also evaluated and was seen to add substantial benefit. Vaccination of 5% of the general population (~350K) in addition to the vaccination of 30% of all HCW was predicted to limit the epidemic to 2,469 cases, equating to a reduction of 57% of the cases compared with vaccination of 30% of HCW alone (Fig 5 and Table 2), and to restrict deaths to 940, a 60% reduction when compared to the scenario wherein only 30% of HCW are vaccinated. Taking this further, vaccination of even 1% of the general population (~70K) in addition to vaccination of 30% of HCW was predicted to result in a reduction of cases and deaths by 15% and 17%, respectively (vs. vaccination of 30% of HCW alone). These benefits of prophylactic vaccination of general population were seen to increase with increase in vaccine coverage, albeit at a lower incremental rate (Fig 6).

Given that i) vaccination of HCW entails only a few hundred vaccinations as against the few hundred thousand vaccinations required to immunize the general population, and ii) the rate of transmission from infected individuals to HCW is significantly higher than to the general population as a result of the more frequent contact of HCW with infected individuals, we examined the relative efficiencies of impact of prophylactic vaccination of HCW and the general population. The numbers of cases averted per vaccination were seen to be 8.47 (interquartile ranges [IQR]:5.03–11.26) with vaccination of 30% of HCW alone, 0.054 (IQR: 0.031–0.073) with vaccination of both 30% of HCW and 1% of the general population, and 0.0178 (IQR: 0.0096–0.0244) with vaccination of both 30% of HCW and 5% of the general population.

**Fig 7:**
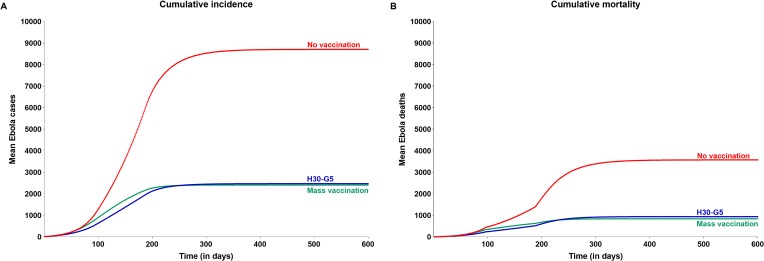
Impact of reactive mass vaccination on cumulative incidence and mortality associated with Ebola virus disease, compared with H30-G5 prophylactic vaccination strategy. Vaccine profile assumed for mass vaccination: Time to onset of protection: 7 days, duration of protection: 180 days, efficacy: 100%. Daily rate at which vaccination is carried out: 0.1% (general population) and 5% (HCW). Abbreviations: HCW, health care worker; H30-G5, vaccination of 30% of all HCW plus 5% of the general population.

As is evident from these results, prophylactic vaccination of a few hundred HCW or a few hundred thousand individuals of the general population may have a significant impact on an Ebola outbreak. However, to achieve a similar impact with reactive mass vaccination, much larger numbers will need to be vaccinated. Reactive mass vaccination initiated 30 days after the onset of an epidemic using a vaccine with 100% efficacy against infection, 180 days of protection, and onset of protection in 7 days is expected to avert 72.3% cases and 76.4% deaths compared with a no-vaccination strategy (Fig 7 and Table 3) but will entail vaccinating about half of the general population (about 3.5 million vaccinations) and 100% of HCW, translating into efficiency of only 0.001813 cases averted per vaccination as compared to 0.0178 with prophylactic vaccination of both 30% of HCW and 5% of general population. This substantial difference between the efficiency of these vaccination strategies can be attributed to the time required to carry out vaccinations of a considerable proportion of the population in case of reactive vaccination, given the infrastructural challenges, and compounded by the time taken for onset of protection. These delays will allow the outbreak to gain ground in the interim.

**Table 3:** Impact of reactive mass vaccination on cumulative incidence and mortality associated with Ebola virus disease.

Parameter	No vaccination	Reactive Mass vaccination*
**Number vaccinated**	**0**	**3,473,139**
Cumulative cases (IQR; 95%CI)	8,706 (4,427–12,059; 478–21,942)	2,408 (1,187–3,332; 187–6,360)
Proportion of cases averted vs no vaccine (IQR; 95%CI)	–	72.3% (72%-73%; 61%-73%)
Cumulative deaths (IQR; 95%CI)	3,575 (1,821–4,950; 179–9,031)	842 (420–1,160; 74–2208)
Proportion of deaths averted vs no vaccine (IQR; 95%CI)	–	76.4% (76%-77%; 59%-77%)

Abbreviations: CI, credible interval; IQR, interquartile range

*Vaccine profile assumed: Time to onset of protection: 7 days, duration of protection: 180 days, efficacy: 100%

Daily rate at which vaccination is carried out: 0.1% (general population) and 5% (HCW)

The results of the ‘no vaccination’ scenario in our model revealed wide dispersion around the mean value owing to stochastic variation. This is reflective of the nature of Ebola outbreaks, with some outbreaks over the years waning quite early on, and others tending to have large breakouts prior to being contained (S2 Fig). Such wide dispersion is also seen in all scenarios with different levels of vaccination of HCW, resulting in a fairly consistent magnitude of impact of vaccination across varying outbreak sizes (S3 Fig), implying that the impact of prophylactic vaccinations, in terms of the proportion of cases averted, will generally hold regardless of the potential size of the outbreak.

By way of a sensitivity analysis around key input parameters, we carried out a Monte Carlo simulation by introducing random variations (within a ±10% range) in three key parameters fitted for the 2014 Sierra Leone epidemic—transmission rates, time to hospitalization, and time from hospitalization to recovery/death (S5 Table). The results of the sensitivity analysis revealed that the magnitude of impact of vaccination was quite consistent across the entire spectrum of 50,000 simulations, confirming the findings of the base model (S4 Fig).

## Discussion

In this study, we show that: i) prophylactic vaccination of only 30% of HCW, representing only 4.9 per 1000 of the total population, could have a significant impact, by averting up to 34% of cases and deaths; ii) vaccination of only 5% of the general population in addition to 30% of HCW will add to the impact, helping avert an incremental 57% of cases and 60% of deaths compared with vaccination of 30% HCW alone; and iii) the impact of such vaccination was consistently applicable in the tested geographical/epidemic settings. The small proportions of populations considered for vaccination in these evaluations demonstrate how even modest levels of fully protective vaccination can be substantially effective, largely through elimination of the high rate of transmission from infected individuals to HCW.

Although completely avoiding the risk of a catastrophic epidemic of Ebola virus disease such as the one in 2014 is desirable, it could require prophylactic vaccination of large populations of high-risk countries, making it challenging to implement. Our analyses provide insights into alternatives that could help substantially to accomplish the goal of protection of vulnerable populations against outbreaks in an efficient manner. Prophylactic vaccination of even small proportions of high-risk populations were estimated to result in substantially superior outcomes compared with vaccination initiated following onset of outbreaks or with no vaccination. Our findings are in agreement with results from other modeling analyses of the impact of vaccination. Bodine and colleagues employed an ordinary differential equations model to conclude that with as little as 36% of the general susceptible population and 95% of all HCW being vaccinated prior to an initial infection, the outbreak is unable to spread through the population [26]. Coltart and colleagues, while assessing the 2014 West Africa epidemic, reported that prophylactic vaccination (with 100% efficacy) of 100% and 75% of HCW will help avert 63% and 36% of overall Ebola cases, respectively [36]. Another study by Xie and colleagues used a modified Susceptible, Exposed, Infective, Hospitalized, Funeral, and Removed (SEIHFR) model to assess the impact of vaccination and determined that a randomized mass vaccination strategy before an Ebola outbreak of 1%, 5%, or 10% of the population would help reduce the final total number of cases by 8.8%, 36.5%, 59.1%, respectively, and the final total number of deaths by 8.2%, 34.2%, 56.2%, respectively [27]. The study did not explicitly assess the impact of vaccination of HCW. A study by Robert and colleagues [25,37] reported that prophylactic vaccination of (with 90% efficacy) 50%, 30%, and 10% of HCW will help avert 58%, 40%, and 15% of overall Ebola cases, respectively, in outbreaks similar to the one in Kikwit, Democratic Republic of Congo (1995). However, the study concluded that while HCWs play an outsized role in some outbreaks, they do not play as much of a role in others. The authors have stated that this variability is based on a combination of factors, but that it cannot be predicted ahead of an actual outbreak what the role of HCW will be.

Kilmarx and colleagues [38], while evaluating the 2014 Sierra Leone epidemic, reported that cumulative incidence in HCW was 8,285 per 100,000, a rate that was 103 times higher than in the general adult population. In our model, cumulative incidence in HCW was 25,672 per 100,000 versus 124 per 100,000 in the general population (i.e., 206 times higher). The significantly greater efficiency in averting cases associated with prophylactically vaccinating HCW as compared to vaccinating the general population is explained by the much higher daily rate of transmission from infected individuals to the susceptible HCW (118 vs 0.635 to the general population).

Affording protection through vaccination of even 1% of the general population, in addition to the vaccination of 30% HCW, prior to the onset of an outbreak will further help avert a large number of cases. While this may not appear very intuitive, it is explained by the exponential nature of infectious disease outbreaks, during which cases multiply rapidly. Each infected individual is at risk of transmitting the disease in each of his/her interactions with individuals who are not protected (e.g., with contacts and contacts of contacts); the next round of infected individuals then goes on to be at similar risk. This cascading effect explains the steep increase in the number of cases averted with incremental protection of the general population. However, also given this exponential nature of transmission of infectious diseases, the rate of decline in cases averted decreases progressively with increasing vaccination coverage (Fig 6).

Protection of 30% of the population including both HCW and the general population prior to the onset of an outbreak would lead to almost complete containment, with only about 135 cases being reported (Fig 6). A 2016 study by Guo and colleagues concluded that immunity in >51% (95% confidence intervals: 44%-56%) of the total population in Sierra Leone would be sufficient to control the epidemic of Ebola virus disease [39]. The more deficient a country’s infrastructure is in its ability to implement speedy reactive vaccination during an outbreak and take other steps necessary to contain the outbreak, the more beneficial prophylactic vaccination can be estimated to be.

The benefits of prophylactic vaccination of HCW also extend beyond those seen directly in the form of reduced Ebola virus disease-related burden. Not vaccinating HCW can have devastatingly negative indirect consequences. A total of 513 HCW, constituting a significant proportion of the local HCW workforce in the affected countries of Guinea, Liberia, and Sierra Leone, died during the 2014 epidemic [33,40]. This made it imperative for many healthcare facilities to close due to paucity of staff [41]. Twenty-five percent of surgeons affiliated with the main teaching hospital in Sierra Leone died during the epidemic in August 2014, resulting in surgical activity falling to a mere 3% of expected levels [42]. Non-availability of adequate numbers of HCW could result in devastating effects on non-Ebola-related health issues in affected countries. A modeling study estimated that these HCW deaths could potentially result in increases in maternal mortality by 38%, 74%, and 111% in Guinea, Sierra Leone, and Liberia, respectively, translating to 4,022 additional maternal deaths [33]. As many as 3.5 million cases of malaria were estimated to have been left untreated and 10,623 additional deaths from HIV/AIDS, tuberculosis, and malaria were estimated to have occurred during and immediately after the epidemic [43,44]. Measles vaccination was estimated to have been missed in a million children, which was estimated to result in an additional 2,000 to 16,000 measles-related deaths in the years to come [45]. Approximately 600,000–700,000 fewer children were estimated to have received important childhood vaccines against diphtheria, tetanus, pertussis, hepatitis B, *Haemophilus influenza* type B, tuberculosis, and polio [45], increasing the likelihood of future increases in morbidity and mortality attributable to these diseases.

Our study has some limitations. First, it is assumed both that the risk of infection is homogeneous across the population and that prophylactic vaccination is carried out at the same rate within the entire general population. In the real world, however, neither assumption regarding homogeneity may hold fully. In such instances, the impact of vaccination as shown in this study may be greater or smaller, although we expect the directionality of this assessment to hold based on results of the various sensitivity analyses shared here. Second, to achieve the levels of protection assumed in the various prophylactic vaccination evaluations, the actual number of vaccinations needed may be higher to account for a) vaccines to be able to provide less than 100% protection against infection, b) waning protection of a vaccine, potentially requiring repetition of vaccination, and c) turnover in HCW staff resulting in new recruits not being immediately protected. Third, the impact of vaccinating HCW will be less pronounced in cases wherein HCW do not play a significant role in an outbreak, as shown in an earlier study [25]. Fourth, the rate of transmission of Ebola infection from dead individuals assumed in the model is a composite of the rate of transmission from community deaths and from deaths in Ebola treatment units (ETUs). Fifth, the estimates of HCW used for disease management in our model are based on the pre-2014 HCW stock reported by the referenced studies and do not include front-line workers or any HCW added to the system during the Ebola outbreak. Sixth, we have not evaluated the impact of ring vaccination explicitly, since 1) the mean-field compartmental model approach is not ideally suited to capture all the key dynamics of ring vaccination, 2) ring vaccination is already established as a go-to strategy in the immediate aftermath of an outbreak, and 3) the impact of ring vaccination implemented in Sierra Leone during the 2014 epidemic has been captured during the fitting process and is thus reflected in the base case. Finally, any births or deaths due to non-Ebola related causes during the evaluation period were not considered.

In conclusion, our study revealed that prophylactic vaccination of even small proportions of HCW and the general population could go a long way towards minimizing the impact of an Ebola virus disease outbreak. We hope these findings will help inform disease control strategies. Further modelling efforts are necessary to fine tune vaccination strategies taking into account the heterogeneity inherent in populations.

## Supporting information

S1 File Methods. (DOCX)

S2 File Supporting data. (XLSX)

S1 Table Search string and inclusion/exclusion criteria. (DOCX)

S2 Table Shortlisted articles. (DOCX)

S3 Table Transitions and the expressions for the stochastic compartmental model. For a pictorial depiction of the transitions, see S5 Fig.
(DOCX)

S4 Table Description of parameters. (DOCX)

S5 Table Sensitivity Analyses to Evaluate the Influence of Variability in Disease Parameters on the Impact of Vaccination of HCW and the General Population. (DOCX)

S1 Fig Schematic of literature search, inclusion, and exclusion of publications. (TIF)

S2 Fig Dispersion in the results of model simulations in the ‘no vaccination’ scenario. The data are based on 5000 simulations.
(TIF)

S3 Fig Dispersion of results of model simulations by evaluation. Results for scenarios in which 10% and 30% of HCW were vaccinated have been depicted.
(TIF)

S4 Fig Sensitivity analyses to evaluate the influence of variability in transmission rates, time to hospitalization, and time from hospitalization to recovery/death on the impact of prophylactic vaccination of HCW and the general population. Monte Carlo simulation was carried out with random variations in these parameters being introduced (within a range of ±10%).
(TIF)

S5 Fig Transitions in the stochastic compartmental model. (TIF)
