# Supplementary material for: Impact of prophylactic vaccination strategies on Ebola virus transmission: A modeling analysis
Source: PLoS One. 2020 Apr 27;15(4):e0230406. doi: 10.1371/journal.pone.0230406 (PMC7185698; doi:10.1371/journal.pone.0230406)
Supplement: S1 File — (DOCX) [file pone.0230406.s001.docx]

# Methods

## **Choice of model**

A literature review was carried out in PubMed, Embase, and the Cochrane Library to identify publications centered on modeling the epidemiology of Ebola infections, with a view to identifying different approaches used for such modeling. The inclusion/exclusion criteria used for this literature review are listed in S1 Table. Additionally, references contained in these identified publications as well as articles found through open searches were included in the exercise. Of the 2,088 publications that were identified through this search, 57 were shortlisted based on the criteria mentioned in S1 Fig. The shortlisted articles are listed in S2 Table. Of the 57 publications reviewed, 43 used a mean-field compartmental model, with the remainder mainly using individual agent-based models. While agent-based models offer greater precision, they require a greater number of inputs and computational power and are therefore not easily replicable, leading us to opt for a mean-field compartmental model. Of the 43 publications based on mean-field compartmental models, 22 used a stochastic approach to estimate and forecast the numbers of cases and deaths in an outbreak, while the rest used deterministic models. Stochastic mean-field compartmental models, which factor in the uncertainty and randomness of events and provide a range of output values, as opposed to the single values provided by deterministic models, are better equipped to handle the uncertain durations and scales of Ebola outbreaks, especially when applied to small subpopulations such as healthcare workers (HCW); we thus chose the stochastic approach to carry out the various evaluations using the model.

## **Ebola transmission model**

A SEIR mean-field compartmental model was used to replicate a past epidemic (the 2014 Ebola epidemic in Sierra Leone). The model was then adapted to incorporate vaccinations and used to evaluate the impact of various vaccination strategies on the cases and deaths due to Ebola. Twelve compartments in the model, as shown in the main text (Fig 1), categorized the total population. The susceptible population was divided into Healthcare Worker (S_HCW_) and General population (S_G_), as the risk of exposure to Ebola virus is higher among healthcare workers. The model allowed for:

- individuals from the susceptible groups to either get exposed (E) to the Ebola virus through an infectious individual [contained in any of hospitalized (H), non-hospitalized (I) or dead but not buried (D) pools], get vaccinated, or remain in the susceptible pool
- exposed individuals to become infectious and move to the ‘Infectious (I)’ compartment where they are capable of infecting others
- infectious individuals to get hospitalized once they are diagnosed. Both the hospitalized and non-hospitalized infectious individuals would either recover on their own and move to the ‘Recovered’ compartment (R) or would die due to disease (D). The dead bodies of these infected individual would continue to infect the susceptible population until burial (B)
- vaccinated individuals to either get exposed to Ebola virus or remain in the ‘Vaccinated’ compartment (from V1 in case of HCW and V3 in case of the general population) until the onset of protection. Those remaining unexposed move to the ‘Protected’ compartment (V2 for HCW and V4 for general population) upon completion of time to onset of protection.

The population (N) of the modeled geography at any given time is the aggregate of populations of all compartments

N = S_G_ + S_HCW_ + V_1_ + V_2_ + V_3_ + V_4_ + E + I + H + D + R+ B

Where, S_G_ = Susceptible general population

S_HCW_ = Susceptible healthcare worker

V_1_ = Vaccinated but not yet protected healthcare worker

V_2_ = Vaccinated and protected healthcare worker

V_3_ = Vaccinated but not yet protected general population

V_4_ = Vaccinated and protected general population

E = Exposed with Ebola virus

I = Infectious with Ebola virus

H = Infectious and Hospitalized

D = Infectious and dead but not buried

R = Recovered from Ebola

B = Dead and buried

At model initiation (t_0_), in the ‘no vaccination’ scenario, the entire population was initially considered susceptible, other than 14 index infectious cases (compartment I). This was in line with the 14 confirmed cases reported by the World Health Organization on May 29, 2014 for the epidemic in Sierra Leone [1]. The population in each individual compartment at any given point in time has been estimated by accounting for the inflows and outflows into the compartment since time t_0_. These flows were captured with the help of the expressions described in S3 Table and the differential equations listed below. The description of the parameters used in these equations are provided in S4 Table. Because of the relatively short time over which the epidemic evolves, we have not considered natural deaths and births in this model.

$$\frac{dS_{G}}{\mathrm{dt}}=-\xi_{2}S_{G}-\left( \frac{\left( I\beta_{I\to NHCW}+{H\beta}_{H\to NHCW}+{D\beta}_{D\to NHCW} \right)}{N} \right)S_{G} S_{G}\left( 0 \right)={S_{G}}_{0}\geq0$$

$$\frac{dS_{\mathrm{HCW}}}{\mathrm{dt}}=-\xi_{1}S_{\mathrm{HCW}}-\left( \frac{\left( I\beta_{I\to HCW}+{H\beta}_{H\to HCW}+{D\beta}_{D\to HCW} \right)}{N} \right)S_{\mathrm{HCW}} S_{\mathrm{HCW}}\left( 0 \right)={S_{\mathrm{HCW}}}_{0}\geq0$$

$$\frac{\mathrm{dE}}{\mathrm{dt}}=\frac{\left( I\beta_{I\to NHCW}+{H\beta}_{H\to NHCW}+{D\beta}_{D\to NHCW} \right)}{N}\left( S_{G}+V_{3} \right)+ \frac{\left( I\beta_{I\to HCW}+{H\beta}_{H\to HCW}+{D\beta}_{D\to HCW} \right)}{N}\left( S_{\mathrm{HCW}}+V_{1} \right)-\sigma E E\left( 0 \right)=E_{0}\geq0$$

$$\frac{\mathrm{dI}}{\mathrm{dt}}=\sigma E-\alpha I-\delta_{1}\gamma I-\left( 1-\delta_{1} \right)\gamma I I\left( 0 \right)=I_{0}\geq0$$

$$\frac{\mathrm{dH}}{\mathrm{dt}}=\alpha I{-(1-\delta}_{2})\gamma_{H}H-\delta_{2}\gamma_{H}H H\left( 0 \right)=H_{0}\geq0$$

$$\frac{\mathrm{dR}}{\mathrm{dt}}=\left( 1-\delta_{1} \right)\gamma I+{(1-\delta}_{2})\gamma_{H}H R\left( 0 \right)=R_{0}\geq0$$

$$\frac{\mathrm{dD}}{\mathrm{dt}}=\delta_{1}\gamma I+\delta_{2}\gamma_{H}H-\gamma_{D}D D\left( 0 \right)=D_{0}\geq0$$

$$\frac{\mathrm{dB}}{\mathrm{dt}}=\gamma_{D}D B\left( 0 \right)=B_{0}\geq0$$

$$\frac{dV_{1}}{\mathrm{dt}}=\xi_{1}S_{\mathrm{HCW}}-\frac{\left( I\beta_{I\to HCW}+{H\beta}_{H\to HCW}+{D\beta}_{D\to HCW} \right)V_{1}}{N}-\phi V_{1} V_{1}\left( 0 \right)={V_{1}}_{0}\geq0$$

$$\frac{dV_{2}}{\mathrm{dt}}=\phi V_{1} V_{2}\left( 0 \right)={V_{2}}_{0}\geq0$$

$$\frac{dV_{3}}{\mathrm{dt}}=\xi_{2}S_{G}-\frac{\left( I\beta_{I\to NHCW}+{H\beta}_{H\to NHCW}+{D\beta}_{D\to NHCW} \right)V_{3}}{N}-\phi V_{3} V_{3}\left( 0 \right)={V_{3}}_{0}\geq0$$

$$\frac{dV_{4}}{\mathrm{dt}}=\phi V_{3} V_{4}\left( 0 \right)={V_{4}}_{0}\geq0$$

For the scenario where reactive mass vaccination strategy with a vaccine of limited durability was assessed, the model was modified to account for transition of patients from the vaccinated and protected pools (V_2_ and V_4_) to the respective susceptible pools (S_HCW_ and S_G_) once the vaccine lost its efficacy after duration τ. The rate of this transition at time t depended on the number of individuals vaccinated at time t – τ. Given this dependency of the transition at time t on the model state at t – τ, where τ is a fixed constant, a fixed time delay, as explained by Zhien Ma and colleagues [2], was included in the equations relevant to S_G_, S_HCW,_ V_2_ and V_4_ compartments as shown below:

1. Susceptible general population (S_G_):

$$\frac{dS_{G}}{\mathrm{dt}}=\left[ 1-\int_{t-\tau}^{t-\tau+\frac{1}{\phi}} \frac{\left( I\left( u \right)\beta_{I\to NHCW}+{H\left( u \right)\beta}_{H\to NHCW}+{D\left( u \right)\beta}_{D\to NHCW} \right)}{N}\mathrm{du} \right]\xi_{2}S_{G}\left( t-\tau\right)-\xi_{23}S_{G}\left( t \right)-\left( \frac{\left( I{\left( t \right)\beta}_{I\to NHCW}+{H\left( t \right)\beta}_{H\to NHCW}+{D\left( t \right)\beta}_{D\to NHCW} \right)}{N} \right)S_{G}\left( t \right)$$

$S_{G}\left( 0 \right)={S_{G}}_{0}\geq0$

The rate of inflow of individuals after the expiration of vaccine efficacy who were vaccinated at t – τ is determined by the number of individuals who were vaccinated at that time, $\xi_{2}S_{G}\left( t-\tau\right)$, as reduced by those that may have been exposed to the virus during the period 1/Ф from the day of vaccination to the onset of vaccine efficacy when the vaccine is yet to offer any protection, captured in the expression

$$\left[ \int_{t-\tau}^{t-\tau+\frac{1}{\phi}} \frac{\left( I\left( u \right)\beta_{I\to NHCW}+{H\left( u \right)\beta}_{H\to NHCW}+{D\left( u \right)\beta}_{D\to NHCW} \right)}{N}\mathrm{du} \right]\xi_{2}S_{G}\left( t-\tau\right)$$

These terms to account for the expiration of vaccine efficacy and consequent return of individuals from the vaccinated compartments to the susceptible compartments has also been inserted in the below three expressions.

1. Susceptible healthcare workers (S_HCW_):

$$\frac{dS_{\mathrm{HCW}}}{\mathrm{dt}}=\left[ 1-\int_{t-\tau}^{t-\tau+\frac{1}{\phi}} \frac{\left( I\left( u \right)\beta_{I\to HCW}+{H\left( u \right)\beta}_{H\to HCW}+{D\left( u \right)\beta}_{D\to HCW} \right)}{N}\mathrm{du} \right]\xi_{1}S_{\mathrm{HCW}}\left( t-\tau\right)-\xi_{1}S_{HCW}\left( t \right)-\left( \frac{\left( I{\left( t \right)\beta}_{I\to HCW}+{H\left( t \right)\beta}_{H\to HCW}+{D\left( t \right)\beta}_{D\to HCW} \right)}{N} \right)S_{HCW}\left( t \right)$$

$S_{\mathrm{HCW}}\left( 0 \right)={S_{\mathrm{HCW}}}_{0}\geq0$

1. Vaccinated and protected healthcare workers (V_2_):

$$\frac{dV_{2}}{\mathrm{dt}}=\phi V_{1}(t)-\left[ 1-\int_{t-\tau}^{t-\tau+\frac{1}{\phi}} \frac{\left( I\left( u \right)\beta_{I\to HCW}+{H\left( u \right)\beta}_{H\to HCW}+{D\left( u \right)\beta}_{D\to HCW} \right)}{N}\mathrm{du} \right]\xi_{1}S_{\mathrm{HCW}}\left( t-\tau\right)$$

$$V_{2}\left( 0 \right)={V_{2}}_{0}\geq0$$

1. Vaccinated and protected general population (V_4_):

$$\frac{dV_{4}}{\mathrm{dt}}=\phi V_{3}\left( t \right)-\left[ 1-\int_{t-\tau}^{t-\tau+\frac{1}{\phi}} \frac{\left( I\left( u \right)\beta_{I\to NHCW}+{H\left( u \right)\beta}_{H\to NHCW}+{D\left( u \right)\beta}_{D\to NHCW} \right)}{N}\mathrm{du} \right]\xi_{2}S_{G}\left( t-\tau\right)$$

$V_{4}\left( 0 \right)={V_{4}}_{0}\geq0$

A deterministic mean-field compartmental model, based on the above equations but excluding terms relating to vaccination, was used to estimate the geographic/epidemic-specific parameters separately for pre-intervention and post-intervention periods using a least square optimization technique. The model equations were also implemented using Gillespie’s direct method algorithm [3] to develop the stochastic mean-field compartmental model which was then used to validate the model and to simulate various vaccine strategies. As part of this algorithm, two random numbers R1 and R2 were generated from the uniform distribution in unit interval. A loop was then initiated involving the following steps: first, the probability $a_{i}$ of each of the n transitions occurring at the current time was calculated; then, the time step to the next event was determined as τ = (1/ a_0_)ln(1/R1) where a_0_ =$\sum_{i=1}^{n} a_{i}$, and the next event E_j_ was determined by the smallest integer j (where 1 < j < n) satisfying $\sum_{i=1}^{j} a_{i}> R_{2}a_{0}$. This next event was then executed by increasing time by τ units and updating the population of the compartments impacted by the event. Finally, the loop was repeated until the end of the model time horizon.

## **Basic reproduction number**

The basic reproduction number (R_0_) was computed for the scenario without any intervention or vaccination (ξ_1_= ξ_2_=0), using a next generation matrix approach suggested by Diekmann and colleagues [4], represented by the equation:

$$R_{0}= \rho\left( {FV}^{-1} \right)$$

Where ρ denotes the spectral radius, and the matrices F and V are transmission (spread of infection) and transition (movement between states) matrices, respectively. The F and V matrices are shown below, where the rows (from top to bottom) and columns (from left to right) represent exposed (E), infected (I), hospitalized (H), and dead (D) individuals, respectively:

Transmission Matrix F Transition Matrix V $\left[ \begin{matrix} . & E & I & H & D \\ E & 0 & {K_{1}\beta}_{I\to\mathrm{HCW}}+\left( {1-K}_{1} \right)\beta_{I\to\mathrm{NHCW}} & {{K_{1}\beta}_{H\to\mathrm{HCW}}+}\left( {1-K}_{1} \right)\beta_{H\to\mathrm{NHCW}} & \beta_{D} \\ I & 0 & 0 & 0 & 0 \\ H & 0 & 0 & 0 & 0 \\ D & 0 & 0 & 0 & 0 \end{matrix} \right] \left[ \begin{matrix} . & E & I & H & D \\ E & \sigma& 0 & 0 & 0 \\ I & -\sigma& \left( \alpha+\gamma\right) & 0 & 0 \\ H & 0 & -\alpha& \gamma_{H} & 0 \\ D & 0 & {-\delta}_{1}\gamma& {-\delta}_{2}\gamma_{H} & \gamma_{D} \end{matrix} \right]$

In transmission matrix F, HCW as a proportion of total population is denoted by K1. The model-fitted transmission rate via contact with infectious individuals was different for HCW (β_I🡪HCW_ in case of infectious individuals not hospitalized, and β_H🡪HCW_ in case of infectious individuals who are hospitalized) and the general population (β_I🡪NHCW_ in case of infectious individuals not hospitalized, and β_H🡪NHCW_ in case of infectious individuals who are hospitalized). However, the transmission rates via contact with dead but not buried (β_D🡪HCW_= β_D🡪NHCW_= β_D_) were the same for both the HCW and general populations.

R_0_ was calculated as follows:

$$R_{0} = \left[ \frac{K_{1}\beta_{I\to HCW}+\left( 1-K_{1} \right)\beta_{I\to NHCW}}{\left( \alpha\right.+\left. \gamma\right)}+\alpha\left( \frac{K_{1}\beta_{H\to HCW}+\left( 1-K_{1} \right)\beta_{H\to NHCW}}{\gamma_{H}\left( \alpha\right.+\left. \gamma\right)} \right)+\frac{\beta_{D}\left( \delta_{1}\gamma+ \left. \delta_{2}\alpha\right) \right.}{\left( \alpha\right.+\left. \gamma\right)\gamma_{D}} \right]$$

$$R_{0}= R_{0I} +R_{0H}+R_{0D}$$

**References**

1. World Health Organization. Ebola virus disease, West Africa – update Geneva, Switzerland2014 [March 18, 2019]. Available from: <https://www.who.int/csr/don/2014_05_28_ebola/en/>.

2. Ma Z, Li J. Dynamical Modeling and Analysis of Epidemics: World Scientific; 2009.

3. Gillespie DT. A general method for numerically simulating the stochastic time evolution of coupled chemical reactions. Journal of Computational Physics. 1976;22(4):403-34. doi: <https://doi.org/10.1016/0021-9991(76)90041-3>.

4. Diekmann O, Heesterbeek JAP, Metz JAJ. On the definition and the computation of the basic reproduction ratio R0 in models for infectious diseases in heterogeneous populations. Journal of mathematical biology. 1990;28(4):365-82. doi: 10.1007/bf00178324.
