# Supplementary material for: Impact of prophylactic vaccination strategies on Ebola virus transmission: A modeling analysis
Source: PLoS One. 2020 Apr 27;15(4):e0230406. doi: 10.1371/journal.pone.0230406 (PMC7185698; doi:10.1371/journal.pone.0230406)
Supplement: S1 Table — (DOCX) [file pone.0230406.s003.docx]

**S1 Table. Search string and inclusion/exclusion criteria.**

| **Search string** |
| --- |
| ((Ebola OR “Ebola virus” OR Ebolavirus OR EBOV OR Filoviridae OR “VP35 protein, filovirus” OR “Ebola Hemorrhagic Fever” OR “Ebola virus disease” OR EVD OR “Ebola virus infection”)) AND (model OR models OR modeling OR modelling) |
| **Inclusion Criteria** |
| - - Studies with epidemiological models on human population affected by Ebola |
| **Exclusion Criteria** |
| - - Studies in animals (primates, monkey, fruit bats and others etc.)   - Case studies, case reports, case series   - Comments, editorials, narratives, letter to editor, opinions   - Studies with generic mathematical models for viral dynamics/transmission dynamics of infectious disease   - Studies with insufficient details on the model used to study transmission dynamics of Ebola   - Publications in language other than English |
