# Supplementary material for: Impact of prophylactic vaccination strategies on Ebola virus transmission: A modeling analysis
Source: PLoS One. 2020 Apr 27;15(4):e0230406. doi: 10.1371/journal.pone.0230406 (PMC7185698; doi:10.1371/journal.pone.0230406)
Supplement: S2 Table — (DOCX) [file pone.0230406.s004.docx]

**S2 Table. Shortlisted articles.**

| **S. No.** | **Author** | **Title** | **Structure of the model** | **Type of model** | **Compartments in the model** |
| --- | --- | --- | --- | --- | --- |
| 1 | Althaus CL et al., 2015 [1] | Ebola virus disease outbreak in Nigeria: Transmission dynamics and rapid control | Mean-field compartmental model | Deterministic | Susceptible, Exposed, Infectious, Recovered, Dead |
| 2 | Astacio J et al., 1996 [2] | Mathematical models to study the outbreaks of Ebola | Mean-field compartmental model | Deterministic | Susceptible, Exposed, Infectious, Recovered |
| 3 | Atangana A et al., 2014 [3] | On the mathematical analysis of Ebola hemorrhagic fever: Deathly infection disease in West African countries | Mean-field compartmental model | Deterministic | Susceptible, Infectious, Recovered, Dead |
| 4 | Agusto FB et al., 2015 [4] | Mathematical assessment of the effect of traditional beliefs and customs on the transmission dynamics of the 2014 Ebola outbreaks | Mean-field compartmental model | Deterministic | Susceptible, Exposed, Symptomatic individuals, Non-hospitalized symptomatic, Hospitalized symptomatic, Recovered individuals, Dead in community, Dead in hospitals, Dead & buried |
| 5 | Barbarossa MV et al., 2015 [5] | Transmission Dynamics and Final Epidemic Size of Ebola Virus Disease Outbreaks with Varying Interventions | Mean-field compartmental model | Deterministic | Susceptible, Exposed, Infectious, Hospitalized, Dead but not buried, Dead and buried, Recovered |
| 6 | Bartlett J et al., 2016 [6] | Mathematical modeling of the 2014/2015 Ebola epidemic in West Africa | Discrete time and age structured model | Deterministic | Not Applicable |
| 7 | Brown GD et al., 2016 [7] | An empirically adjusted approach to reproductive number estimation for stochastic compartmental models: A case study of two Ebola outbreaks | Mean-field compartmental model | Stochastic | Susceptible, Exposed, Infectious, Recovered |
| 8 | Browne C et al., 2015 [8] | Modeling contact tracing in outbreaks with application to Ebola | Mean-field compartmental model | Deterministic | Susceptible, Exposed, Infectious and hospitalized, Infectious and remain in community, Recovered in Hospital, Dead and buried, Recovered, Dead |
| 9 | Camacho A et al., 2014 [9] | Potential for large outbreaks of Ebola virus disease | Mean-field compartmental model | Stochastic | Susceptible, Exposed via hospitals, Exposed via community, Infectious, Hospitalized, Recovered, Dead (but not buried), Buried |
| 10 | Camacho A et al., 2015 [10] | Estimating the probability of demonstrating vaccine efficacy in the declining Ebola epidemic: a Bayesian modelling approach | Mean-field compartmental model | Stochastic | Susceptible, Vaccinated but susceptible, Vaccinated and protected, Controlled, Exposed, Infectious, Removed |
| 11 | Chowell G et al., 2005 [11] | Mathematical models of emergent and re-emergent infectious diseases: Assessing the effects of public health interventions on disease spread | Mean-field compartmental model | Deterministic & Stochastic | Susceptible, Exposed, Infectious, Recovered |
| 12 | Cope RC et al., 2014 [12] | Assessment of the Risk of Ebola Importation to Australia | Mean-field compartmental model | Stochastic | Susceptible, Exposed, Infectious, Recovered |
| 13 | Diakite I et al., 2016 [13] | Novel Ordered Stepped-Wedge Cluster trial designs for detecting Ebola vaccine efficacy using a spatially structured mathematical model | Mean-field compartmental model | Stochastic | Susceptible, Exposed, Infectious with dry symptoms in community, Infectious with dry symptoms at ETU, Infectious with wet symptoms in community, Infectious with wet symptoms at ETU, Dead but not buried, Dead and buried, Recovered |
| 14 | Do TS et al., 2016 [14] | Modeling the spread of Ebola | Mean-field compartmental model | Stochastic | Susceptible, Infectious, Latent, Dead and buried, Recovered |
| 15 | Drake JM et al., 2015 [15] | Ebola Cases and Health System Demand in Liberia | Mean-field compartmental model | Stochastic | Community, Hospital, Community treated patients, Hospital treated patient, Health care workers, Visitors to hospital |
| 16 | Evans RJ et al., 2015 [16] | Dynamics of Ebola epidemics in West Africa 2014 | Mean-field compartmental model | Deterministic | Infectious, Recovered |
| 17 | Fast SM et al., 2015 [17] | The Role of Social Mobilization in Controlling Ebola Virus in Lofa County, Liberia | Agent based network model | Stochastic | Susceptible, Exposed, Infectious, Hospitalized, Dead but not buried, Dead and buried, Recovered |
| 18 | Feng Z et al., 2016 [18] | Mathematical models of Ebola—Consequences of underlying assumptions | Mean-field compartmental model | Stochastic | Susceptible, Exposed, Infectious, Hospitalized, Recovered, Dead |
| 19 | Frasso G et al., 2016 [19] | Bayesian inference in an extended SEIR model with nonparametric disease transmission rate: an application to the Ebola epidemic in Sierra Leone | Mean-field compartmental model | Stochastic | Susceptible, Exposed, Infectious, Recovered, Dead |
| 20 | Gomes MFC et al, 2014 [20] | Assessing the International Spreading Risk Associated with the 2014 West African Ebola Outbreak | Mobility network model: Global Epidemic and Mobility (GLEaM) model | Stochastic | Susceptible, Exposed, Infectious, Hospitalized, Dead but not buried, Dead and buried, Recovered |
| 21 | Guo Z et al., 2016 [21] | Predicting and evaluating the epidemic trend of Ebola Virus Disease in the 2014-2015 outbreak and the effects of intervention measures | Mean-field compartmental model | Stochastic | Susceptible, Suspected, Observation, Confirmed cases in isolation, Hospital staff, Exposed, Infectious, Dead but not buried, Recovering but still infectious, Recovering and non-infectious |
| 22 | Ivorra B et al., 2015 [22] | Be-CoDiS: A mathematical model to predict the risk of human diseases spread between countries—Validation and application to the 2014–2015 Ebola virus disease epidemic | Mean-field compartmental model | Deterministic | Susceptible, Exposed, Infectious, Recovered, Dead but not buried, Dead and buried |
| 23 | Khan A et al., 2015 [23] | Estimating the basic reproductive ratio for the Ebola outbreak in Liberia and Sierra Leone | Mean-field compartmental model | Deterministic | High risk susceptible, Low risk susceptible, Exposed, Infectious, Hospitalized, Recovered |
| 24 | King AA et al., 2015 [24] | Avoidable errors in the modeling of outbreaks of emerging pathogens, with special reference to Ebola | Mean-field compartmental model | Stochastic | Susceptible, Exposed, Infectious, Recovered |
| 25 | Kiskowski MA et al., 2014 [25] | A Three Scale Network Model for the early growth dynamics of 2014 West Africa Ebola epidemic | Agent based network model | Stochastic | Susceptible, Exposed, Infectious, Recovered |
| 26 | Kiskowski M et al., 2015 [26] | [Modeling household and community transmission of Ebola virus disease: epidemic growth, spatial dynamics and insights for epidemic control](#RANGE!C14) | Agent based network model | Stochastic | Susceptible, Exposed, Infectious, Recovered |
| 27 | Kucharski AJ et al., 2015 [27] | Evaluation of the benefits and risks of introducing Ebola Community Care Centers, Sierra Leone | Mean-field compartmental model | Deterministic | Susceptible, Exposed, Infectious with Ebola like symptoms, Infectious with Ebola symptoms, Recovered, Ebola treatment center/ Community care center |
| 28 | Kucharski AJ et al., 2015 [28] | Measuring the impact of Ebola control measures in Sierra Leone | Mean-field compartmental model | Stochastic | Susceptible, Exposed, Reported Infectious, Missed Infectious, Ebola health care center/ Community care center, Ebola treatment unit, Recovered |
| 29 | Kucharski AJ et al., 2016 [29] | Effectiveness of Ring Vaccination as control strategy for Ebola virus disease | Agent based network model | Stochastic | Exposed, Infectious, Recovered |
| 30 | Leander RN et al., 2016 [30] | Modeling Ebola within a community | Mean-field compartmental model | Deterministic | Susceptible, Exposed, Infectious, Recovered, Dead |
| 31 | Legrand J et al., 2007 [31] | Understanding the dynamics of Ebola epidemics | Mean-field compartmental model | Stochastic | Susceptible, Exposed, Infectious, Recovered |
| 32 | Ndanusa A et al., 2015 [32] | A mathematical model for controlling the spread of Ebola virus disease in Nigeria | Mean-field compartmental model | Deterministic | Susceptible, Latent, Infectious, Recovered |
| 33 | Ngwa GA et al., 2016 [33] | A mathematical model with quarantine states for the dynamics of Ebola virus disease in human populations | Mean-field compartmental model | Deterministic | Susceptible, Suspected non-quarantined cases, Suspected quarantined cases, Probable non-quarantined cases, Probable quarantined cases, Confirmed non-quarantined early symptomatic cases, Confirmed quarantined early symptomatic cases, Confirmed non-quarantined late symptomatic cases, Confirmed quarantined late symptomatic cases, Recovered, Dead but not buried |
| 34 | Rachah A et al., 2015 [34] | Mathematical modeling, simulation, and optimal control of the 2014 Ebola outbreak in West Africa | Mean-field compartmental model | Deterministic | Susceptible, Infectious, Recovered |
| 35 | Rivers CM et al., 2014 [35] | Modeling the impact of interventions on an epidemic of Ebola in Sierra Leone and Liberia | Mean-field compartmental model | Stochastic | Susceptible, Exposed, Infectious, Hospitalized, Dead but not buried, Recovered |
| 36 | Rizzo A et al., 2016 [36] | [A network model for Ebola spreading](#RANGE!C24) | Activity driven network model | Stochastic | Susceptible, Exposed, Infectious, Hospitalized, Dead but not buried, Dead and buried, Recovered |
| 37 | Shaman J et al., 2014 [37] | Inference and forecast of the current West African Ebola outbreak in Guinea, Sierra Leone and Liberia | Mean-field compartmental model | Stochastic | Susceptible, Exposed, Infectious, Recovered, Dead |
| 38 | Shen M et al., 2015 [38] | Modeling the effect of comprehensive interventions on Ebola virus transmission | Mean-field compartmental model | Deterministic | Susceptible, Vaccinated, Latent undetectable, Latent detectable, Infectious, Isolated, Dead but not buried, Recovered |
| 39 | Siettos CI et al., 2016 [39] | [Forecasting and control policy assessment for the Ebola virus disease (EVD) epidemic in Sierra Leone using small-world networked model simulations](#RANGE!C2) | Agent based network model | Stochastic | Susceptible, Exposed, Infectious, Recovered, Dead but not buried, Dead and buried |
| 40 | Siettos C et al., 2015 [40] | Modeling the 2014 Ebola virus epidemic – Agent based simulations, temporal analysis and future predictions for Liberia and Sierra Leone | Agent based network model | Stochastic | Susceptible, Exposed, Infectious, Recovered, Dead, Buried, Unburied |
| 41 | Valdez LD et al., 2015 [41] | Predicting the extinction of Ebola spreading in Liberia due to mitigation strategies | Mean-field compartmental model | Stochastic | Susceptible, Exposed, Infectious who will be cured without hospitalization, Infectious who will be cured in a hospital, Infectious who will die without hospitalization, Infectious who will die in hospital, Dead but not buried, Recovered |
| 42 | Webb GF et al., 2016 [42] | A model of the Ebola epidemics in West Africa incorporating age of infection | Agent based network model | Stochastic | Susceptible, Infectious, Recovered |
| 43 | Webb G et al., 2015 [43] | A model of the 2014 Ebola epidemic in West Africa with contact tracing | Mean-field compartmental model | Stochastic | Susceptible, Exposed, Infectious, Recovered, Dead |
| 44 | Weitz JS et al., 2015 [44] | Modeling Post-death transmission of Ebola: Challenges for inference and opportunities for control | Mean-field compartmental model | Stochastic | Susceptible, Exposed, Infectious, Recovered, Dead |
| 45 | White RA et al., 2015 [45] | Projected Treatment Capacity Needs in Sierra Leone | Mean-field compartmental model | Stochastic | Susceptible, Exposed, Infectious, Treated-reported, Recovered-unreported, Dead-unreported, Recovered-reported, Dead-reported |
| 46 | Xia ZQ et al., 2015 [46] | Modeling the transmission dynamics of Ebola virus disease in Liberia | Mean-field compartmental model | Deterministic | Susceptible, Exposed, Probable cases, Suspected cases, Recovered |
| 47 | Yamin D et al., 2015 [47] | Effect of Ebola Progression on Transmission and Control in Liberia | Agent based network model | Stochastic | Incubation, Early symptomatic, Late symptomatic |
| 48 | Li Z et al., 2015 [48] | Dynamical analysis of an SEIT epidemic model with application to Ebola virus transmission in Guinea | Mean-field compartmental model | Stochastic | Susceptible, Exposed, Infectious, Recovered |
| 49 | Zhu JM et al., 2016 [49] | Eradication of Ebola based on dynamic programming | Mean-field compartmental model | Deterministic | Susceptible, Exposed, Infectious, Recovered |
| 50 | Merler S et al., 2015 [50] | Spatiotemporal spread of the 2014 outbreak of Ebola virus disease in Liberia and the effectiveness of non-pharmaceutical interventions: a computational modelling analysis | Agent based network model | Stochastic | Susceptible, Exposed, Infectious, Hospitalized, Dead but not buried, Recovered |
| 51 | Ajelli M et al., 2016 [51] | Spatiotemporal dynamics of the Ebola epidemic in Guinea and implications for vaccination and disease elimination: a computational modeling analysis | Agent based network model | Stochastic | Susceptible, Vaccinated but susceptible, Vaccinated and protected, Exposed, Infectious, Hospitalized, Dead but not buried, Recovered |
| 52 | Fasina FO et al., 2014 [52] | Transmission dynamics and control of Ebola virus disease outbreak in Nigeria, July to September 2014 | Mean-field compartmental model | Stochastic | Susceptible, Exposed, Infectious, Hospitalized, Recovered |
| 53 | Wiratsudakul A. et al., 2016 [53] | A one-year effective reproduction number of the 2014-2015 Ebola outbreaks in the widespread West African countries and quantitative evaluation of air travel restriction measure | Mean-field compartmental model | Stochastic | Susceptible, Infectious, Recovered |
| 54 | Lekone PE et al., 2006 [54] | Statistical Inference in a Stochastic Epidemic SEIR Model with Control Intervention: Ebola as a Case Study | Mean-field compartmental model | Stochastic | Susceptible, Exposed, Infectious, Recovered |
| 55 | Camacho A et al., 2015 [55] | Temporal Changes in Ebola Transmission in Sierra Leone and Implications for Control Requirements: A Real time Modelling Study | Mean-field compartmental model | Stochastic | Susceptible, Exposed, Infectious, Hospitalization, Recovered |
| 56 | Lewnard JA et al., 2014 [56] | Dynamics and control of Ebola virus transmission in Montserrado, Liberia: a mathematical modelling analysis | Mean-field compartmental model | Stochastic | Susceptible, Exposed, Infectious but recovering, Infectious not recovering, Ascertained infectious, Dead but not buried, Recovered |
| 57 | Meltzer MI et al., 2014 [57] | Estimating the Future Number of Cases in the Ebola Epidemic— Liberia and Sierra Leone, 2014–2015 | Mean-field compartmental model | Deterministic | Susceptible, Exposed, Incubating, Infectious, Hospitalized, Dead but not buried, Recovered |

**References**

1. Althaus CL, Low N, Musa EO, Shuaib F, Gsteiger S. Ebola virus disease outbreak in Nigeria: Transmission dynamics and rapid control. Epidemics. 2015;11:80-4. Epub 2015/03/21. doi: 10.1016/j.epidem.2015.03.001.

2. Astacio J, Briere D, Guillen M, Martinez J, Rodriguez F, Valenzuela-Campos N. Mathematical models to study the outbreaks of Ebola. 1996.

3. Atangana A, Goufo EFD. On the mathematical analysis of Ebola hemorrhagic fever: deathly infection disease in West African countries. Biomed Res Int. 2014;2014:261383-. Epub 2014/09/11. doi: 10.1155/2014/261383.

4. Agusto FB, Teboh-Ewungkem MI, Gumel AB. Mathematical assessment of the effect of traditional beliefs and customs on the transmission dynamics of the 2014 Ebola outbreaks. BMC medicine. 2015;13:96-. doi: 10.1186/s12916-015-0318-3.

5. Barbarossa MV, Dénes A, Kiss G, Nakata Y, Röst G, Vizi Z. Transmission Dynamics and Final Epidemic Size of Ebola Virus Disease Outbreaks with Varying Interventions. PloS one. 2015;10(7):e0131398-e. doi: 10.1371/journal.pone.0131398.

6. Bartlett J, Devinney J, Pudlowski E. Mathematical modeling of the 2014/2015 Ebola epidemic in West Africa. SIAM Undergraduate Research Online. 2016;9:87-102.

7. Brown GD, Oleson JJ, Porter AT. An empirically adjusted approach to reproductive number estimation for stochastic compartmental models: A case study of two Ebola outbreaks. Biometrics. 2016;72(2):335-43. Epub 2015/11/18. doi: 10.1111/biom.12432.

8. Browne C, Gulbudak H, Webb G. Modeling contact tracing in outbreaks with application to Ebola. J Theor Biol. 2015;384:33-49. Epub 2015/08/18. doi: 10.1016/j.jtbi.2015.08.004.

9. Camacho A, Kucharski AJ, Funk S, Breman J, Piot P, Edmunds WJ. Potential for large outbreaks of Ebola virus disease. Epidemics. 2014;9:70-8. Epub 2014/12/07. doi: 10.1016/j.epidem.2014.09.003.

10. Camacho A, Eggo RM, Funk S, Watson CH, Kucharski AJ, Edmunds WJ. Estimating the probability of demonstrating vaccine efficacy in the declining Ebola epidemic: a Bayesian modelling approach. BMJ open. 2015;5(12):e009346. Epub 2015/12/17. doi: 10.1136/bmjopen-2015-009346.

11. Chowell-Punte G. Mathematical Models of Emergent and Re-emergent Infectious Diseases: Assessing the Effects of Public Health Interventions on Disease Spread: Cornell University, Jan.; 2005.

12. Cope RC, Cassey P, Hugo GJ, Ross JV. Assessment of the Risk of Ebola Importation to Australia. PLoS Curr. 2014;6. Epub 2015/02/17. doi: 10.1371/currents.outbreaks.aa0375fd48a92c7c9422aa543a88711f.

13. Diakite I, Mooring EQ, Velasquez GE, Murray MB. Novel Ordered Stepped-Wedge Cluster Trial Designs for Detecting Ebola Vaccine Efficacy Using a Spatially Structured Mathematical Model. PLoS neglected tropical diseases. 2016;10(8):e0004866. Epub 2016/08/11. doi: 10.1371/journal.pntd.0004866.

14. Do TS, Lee YS. Modeling the Spread of Ebola. Osong public health and research perspectives. 2016;7(1):43-8. Epub 2016/01/04. doi: 10.1016/j.phrp.2015.12.012.

15. Drake JM, Kaul RB, Alexander LW, O'Regan SM, Kramer AM, Pulliam JT, et al. Ebola Cases and Health System Demand in Liberia. PLoS biology. 2015;13(1):e1002056. Epub 2015/01/15. doi: 10.1371/journal.pbio.1002056.

16. Evans RJ, Mammadov M. Dynamics of Ebola epidemics in West Africa 2014. F1000Res. 2014;3:319-. doi: 10.12688/f1000research.5941.2.

17. Fast SM, Mekaru S, Brownstein JS, Postlethwaite TA, Markuzon N. The Role of Social Mobilization in Controlling Ebola Virus in Lofa County, Liberia. PLoS currents. 2015;7:ecurrents.outbreaks.c3576278c66b22ab54a25e122fcdbec1. doi: 10.1371/currents.outbreaks.c3576278c66b22ab54a25e122fcdbec1.

18. Feng Z, Zheng Y, Hernandez-Ceron N, Zhao H, Glasser JW, Hill AN. Mathematical models of Ebola-Consequences of underlying assumptions. Math Biosci. 2016;277:89-107. Epub 2016/04/26. doi: 10.1016/j.mbs.2016.04.002.

19. Frasso G, Lambert P. Bayesian inference in an extended SEIR model with nonparametric disease transmission rate: an application to the Ebola epidemic in Sierra Leone. Biostatistics (Oxford, England). 2016;17(4):779-92. Epub 2016/06/22. doi: 10.1093/biostatistics/kxw027.

20. Gomes MFC, Pastore Y Piontti A, Rossi L, Chao D, Longini I, Halloran ME, et al. Assessing the International Spreading Risk Associated with the 2014 West African Ebola Outbreak. PLoS currents. 2014;6:ecurrents.outbreaks.cd818f63d40e24aef769dda7df9e0da5. doi: 10.1371/currents.outbreaks.cd818f63d40e24aef769dda7df9e0da5.

21. Guo Z, Xiao D, Li D, Wang X, Wang Y, Yan T, et al. Predicting and evaluating the epidemic trend of Ebola Virus Disease in the 2014-2015 outbreak and the effects of intervention measures. PloS one. 2016;11(4):e0152438. Epub 2016/04/07. doi: 10.1371/journal.pone.0152438.

22. Ivorra B, Ngom D, Ramos ÁM. Be-CoDiS: A mathematical model to predict the risk of human diseases spread between countries--validation and application to the 2014-2015 Ebola virus disease epidemic. Bull Math Biol. 2015;77(9):1668-704. Epub 2015/10/08. doi: 10.1007/s11538-015-0100-x.

23. Khan A, Naveed M, Dur-E-Ahmad M, Imran M. Estimating the basic reproductive ratio for the Ebola outbreak in Liberia and Sierra Leone. Infect Dis Poverty. 2015;4:13-. doi: 10.1186/s40249-015-0043-3.

24. King AA, Domenech de Celles M, Magpantay FM, Rohani P. Avoidable errors in the modelling of outbreaks of emerging pathogens, with special reference to Ebola. Proceedings Biological sciences. 2015;282(1806):20150347. Epub 2015/04/04. doi: 10.1098/rspb.2015.0347.

25. Kiskowski MA. A Three-Scale Network Model for the early growth dynamics of 2014 West Africa Ebola epidemic. PLoS currents. 2014;6:ecurrents.outbreaks.c6efe8274dc55274f05cbcb62bbe6070. doi: 10.1371/currents.outbreaks.c6efe8274dc55274f05cbcb62bbe6070.

26. Kiskowski M, Chowell G. Modeling household and community transmission of Ebola virus disease: Epidemic growth, spatial dynamics and insights for epidemic control. Virulence. 2016;7(2):163-73. Epub 2015/08/20. doi: 10.1080/21505594.2015.1076613.

27. Kucharski AJ, Camacho A, Checchi F, Waldman R, Grais RF, Cabrol J-C, et al. Evaluation of the benefits and risks of introducing Ebola community care centers, Sierra Leone. Emerging infectious diseases. 2015;21(3):393-9. doi: 10.3201/eid2103.141892.

28. Kucharski AJ, Camacho A, Flasche S, Glover RE, Edmunds WJ, Funk S. Measuring the impact of Ebola control measures in Sierra Leone. Proceedings of the National Academy of Sciences of the United States of America. 2015;112(46):14366-71. Epub 2015/10/16. doi: 10.1073/pnas.1508814112.

29. Kucharski AJ, Eggo RM, Watson CH, Camacho A, Funk S, Edmunds WJ. Effectiveness of Ring Vaccination as Control Strategy for Ebola Virus Disease. Emerging infectious diseases. 2016;22(1):105-8. doi: 10.3201/eid2201.151410.

30. Leander RN, Goff WS, Murphy CW, Pulido SA. Modelling Ebola within a community. Epidemiology and infection. 2016;144(11):2329-37. Epub 2016/03/28. doi: 10.1017/S0950268816000558.

31. Legrand J, Grais RF, Boelle PY, Valleron AJ, Flahault A. Understanding the dynamics of Ebola epidemics. Epidemiology and infection. 2007;135(4):610-21. Epub 2006/09/27. doi: 10.1017/s0950268806007217.

32. Ndanusa A, Abdulrahman S, Abdulmalik A. A mathematical model for controlling the spread of Ebola virus disease in Nigeria. Int J Human Manage Sci. 2015;3(3).

33. Ngwa GA, Teboh-Ewungkem MI. A mathematical model with quarantine states for the dynamics of Ebola virus disease in human populations. Computational and mathematical methods in medicine. 2016;2016:9352725-. Epub 2016/08/07. doi: 10.1155/2016/9352725.

34. Rachah A, Torres DFM. Mathematical modelling, simulation, and optimal control of the 2014 Ebola outbreak in West Africa. Discrete Dynamics in Nature and Society. 2015;2015.

35. Rivers CM, Lofgren ET, Marathe M, Eubank S, Lewis BL. Modeling the impact of interventions on an epidemic of Ebola in Sierra Leone and Liberia. PLoS Curr. 2014;6. Epub 2015/02/17. doi: 10.1371/currents.outbreaks.4d41fe5d6c05e9df30ddce33c66d084c.

36. Rizzo A, Pedalino B, Porfiri M. A network model for Ebola spreading. J Theor Biol. 2016;394:212-22. Epub 2016/01/21. doi: 10.1016/j.jtbi.2016.01.015.

37. Shaman J, Yang W, Kandula S. Inference and forecast of the current west african ebola outbreak in Guinea, sierra leone and liberia. PLoS Curr. 2014;6. Epub 2015/02/03. doi: 10.1371/currents.outbreaks.3408774290b1a0f2dd7cae877c8b8ff6.

38. Shen M, Xiao Y, Rong L. Modeling the effect of comprehensive interventions on Ebola virus transmission. Scientific reports. 2015;5:15818-. doi: 10.1038/srep15818.

39. Siettos CI, Anastassopoulou C, Russo L, Grigoras C, Mylonakis E. Forecasting and control policy assessment for the Ebola virus disease (EVD) epidemic in Sierra Leone using small-world networked model simulations. BMJ open. 2016;6(1):e008649-e. doi: 10.1136/bmjopen-2015-008649.

40. Siettos C, Anastassopoulou C, Russo L, Grigoras C, Mylonakis E. Modeling the 2014 Ebola virus epidemic - agent-based simulations, temporal analysis and future predictions for liberia and sierra leone. PLoS currents. 2015;7:ecurrents.outbreaks.8d5984114855fc425e699e1a18cdc6c9. doi: 10.1371/currents.outbreaks.8d5984114855fc425e699e1a18cdc6c9.

41. Valdez LD, Aragao Rego HH, Stanley HE, Braunstein LA. Predicting the extinction of Ebola spreading in Liberia due to mitigation strategies. Scientific reports. 2015;5:12172. Epub 2015/07/21. doi: 10.1038/srep12172.

42. Webb GF, Browne CJ. A model of the Ebola epidemics in West Africa incorporating age of infection. Journal of biological dynamics. 2016;10:18-30. doi: 10.1080/17513758.2015.1090632.

43. Webb G, Browne C, Huo X, Seydi O, Seydi M, Magal P. A model of the 2014 ebola epidemic in west Africa with contact tracing. PLoS Curr. 2015;7. Epub 2015/02/17. doi: 10.1371/currents.outbreaks.846b2a31ef37018b7d1126a9c8adf22a.

44. Weitz JS, Dushoff J. Modeling post-death transmission of Ebola: challenges for inference and opportunities for control. Scientific reports. 2015;5:8751. Epub 2015/03/05. doi: 10.1038/srep08751.

45. White RA, MacDonald E, de Blasio BF, Nygard K, Vold L, Rottingen JA. Projected treatment capacity needs in Sierra Leone. PLoS Curr. 2015;7. Epub 2015/02/17. doi: 10.1371/currents.outbreaks.3c3477556808e44cf41d2511b21dc29f.

46. Xia Z-Q, Wang S-F, Li S-L, Huang L-Y, Zhang W-Y, Sun G-Q, et al. Modeling the transmission dynamics of Ebola virus disease in Liberia. Scientific reports. 2015;5:13857-. doi: 10.1038/srep13857.

47. Yamin D, Gertler S, Ndeffo-Mbah ML, Skrip LA, Fallah M, Nyenswah TG, et al. Effect of Ebola progression on transmission and control in Liberia. Ann Intern Med. 2015;162(1):11-7. doi: 10.7326/M14-2255.

48. Li Z, Teng Z, Feng X, Li Y, Zhang H. Dynamical Analysis of an SEIT Epidemic Model with Application to Ebola Virus Transmission in Guinea. Computational and mathematical methods in medicine. 2015;2015:582625. Epub 2015/08/08. doi: 10.1155/2015/582625.

49. Zhu J-M, Wang L, Liu J-B. Eradication of Ebola Based on Dynamic Programming. Computational and mathematical methods in medicine. 2016;2016:1580917-. Epub 2016/05/25. doi: 10.1155/2016/1580917.

50. Merler S, Ajelli M, Fumanelli L, Gomes MFC, Piontti APY, Rossi L, et al. Spatiotemporal spread of the 2014 outbreak of Ebola virus disease in Liberia and the effectiveness of non-pharmaceutical interventions: a computational modelling analysis. The Lancet Infectious diseases. 2015;15(2):204-11. Epub 2015/01/07. doi: 10.1016/S1473-3099(14)71074-6.

51. Ajelli M, Merler S, Fumanelli L, Pastore Y Piontti A, Dean NE, Longini IM, Jr., et al. Spatiotemporal dynamics of the Ebola epidemic in Guinea and implications for vaccination and disease elimination: a computational modeling analysis. BMC medicine. 2016;14(1):130-. doi: 10.1186/s12916-016-0678-3.

52. Fasina FO, Shittu A, Lazarus D, Tomori O, Simonsen L, Viboud C, et al. Transmission dynamics and control of Ebola virus disease outbreak in Nigeria, July to September 2014. Euro surveillance : bulletin Europeen sur les maladies transmissibles = European communicable disease bulletin. 2014;19(40):20920. Epub 2014/10/18.

53. Wiratsudakul A, Triampo W, Laosiritaworn Y, Modchang C. A one-year effective reproduction number of the 2014-2015 Ebola outbreaks in the widespread West African countries and quantitative evaluation of air travel restriction measure. Travel medicine and infectious disease. 2016;14(5):481-8. Epub 2016/10/25. doi: 10.1016/j.tmaid.2016.06.011.

54. Lekone PE, Finkenstadt BF. Statistical inference in a stochastic epidemic SEIR model with control intervention: Ebola as a case study. Biometrics. 2006;62(4):1170-7. Epub 2006/12/13. doi: 10.1111/j.1541-0420.2006.00609.x.

55. Camacho A, Kucharski A, Aki-Sawyerr Y, White MA, Flasche S, Baguelin M, et al. Temporal Changes in Ebola Transmission in Sierra Leone and Implications for Control Requirements: a Real-time Modelling Study. PLoS Curr. 2015;7. Epub 2015/03/05. doi: 10.1371/currents.outbreaks.406ae55e83ec0b5193e30856b9235ed2.

56. Lewnard JA, Ndeffo Mbah ML, Alfaro-Murillo JA, Altice FL, Bawo L, Nyenswah TG, et al. Dynamics and control of Ebola virus transmission in Montserrado, Liberia: a mathematical modelling analysis. The Lancet Infectious diseases. 2014;14(12):1189-95. Epub 2014/12/03. doi: 10.1016/s1473-3099(14)70995-8.

57. Meltzer MI, Atkins CY, Santibanez S, Knust B, Petersen BW, Ervin ED, et al. Estimating the future number of cases in the Ebola epidemic--Liberia and Sierra Leone, 2014-2015. MMWR Suppl. 2014;63(3):1-14.
