# Supplementary material for: Impact of prophylactic vaccination strategies on Ebola virus transmission: A modeling analysis
Source: PLoS One. 2020 Apr 27;15(4):e0230406. doi: 10.1371/journal.pone.0230406 (PMC7185698; doi:10.1371/journal.pone.0230406)
Supplement: S3 Table — For a pictorial depiction of the transitions, see S5 Fig. (DOCX) [file pone.0230406.s005.docx]

**S3 Table. Transitions and expressions in the stochastic compartmental model.** For a pictorial depiction of the transitions, see S5 Fig.

| **#** | **Transition** | **Expressions** | **Transition due to:** |
| --- | --- | --- | --- |
| 1 | (S_HCW_, E) → (S_HCW_ − 1, E + 1) | $\left( \frac{\left( I{\left( t \right)\beta}_{I\to HCW}+{H\left( t \right)\beta}_{H\to HCW}+{D\left( t \right)\beta}_{D\to HCW} \right)}{N} \right)S_{\mathrm{HCW}}$ | Infection of healthcare workers by infected, hospitalized, and dead but not buried individuals |
| 2 | (S_G_, E) → (S_G_ − 1, E + 1) | $\left( \frac{\left( I{\left( t \right)\beta}_{I\to NHCW}+{H\left( t \right)\beta}_{H\to NHCW}+{D\left( t \right)\beta}_{D\to\mathrm{NHCW}} \right)}{N} \right)S_{G}$ | Infection of the general population (non-healthcare workers) by infected, hospitalized, and dead but not buried individuals |
| 3 | (S_HCW_, V_1_) → (S_HCW_ − 1, V_1_ + 1) | $\xi_{1}S_{\mathrm{HCW}}$ | Vaccination of healthcare workers |
| 4 | (S_G_, V_3_) → (S_G_ − 1, V_3_ + 1) | $\xi_{2}S_{G}$ | Vaccination of the general population |
| 5 | (V_1_, V_2_) → (V_1_ -1, V_2_ +1) | $\phi V_{1}$ | Onset of efficacy of vaccine |
| 6 | (V_3_, V_4_) → (V_3_ -1, V_4_ +1) | $\phi V_{3}$ | Onset of efficacy of vaccine |
| 7 | (V_1_, E) → (V_1_ -1, E +1) | $\frac{\left( I\beta_{I\to HCW}+{H\beta}_{H\to HCW}+{D\beta}_{D\to HCW} \right)V_{1}}{N}$ | Infection of vaccinated healthcare workers before onset of efficacy by infected, hospitalized, and dead but not buried individuals |
| 8 | (V_3_, E) → (V_3_ -1, E +1) | $\frac{\left( I\beta_{I\to NHCW}+{H\beta}_{H\to NHCW}+{D\beta}_{D\to NHCW} \right)V_{3}}{N}$ | Infection of the general population (non-healthcare workers) before onset of efficacy by infected, hospitalized, and dead but not buried individuals |
| 9 | (E, I) → (E−1, I + 1) | σE | Onset of infectiousness after completion of the latency period |
| 10 | (I, H) → (I−1, H + 1) | αI | Hospitalization of infectious individuals |
| 11 | (I, R) → (I−1, R + 1) | $\left( 1-\delta_{1} \right)\gamma I$ | Self-recovery of infectious individuals from disease |
| 12 | (I, D) → (I−1, D + 1) | $\delta_{1}\gamma I$ | Death of infectious individuals before hospitalization |
| 13 | (H, R) → (H−1, R + 1) | ${(1-\delta}_{2})\gamma_{H}H$ | Recovery of infectious individuals from disease after hospitalization |
| 14 | (H, D) → (H−1, D + 1) | $\delta_{2}\gamma_{H}H$ | Death during hospital stay |
| 15 | (D, B) → (D−1, B + 1) | $\gamma_{D}D$ | Burial/isolation of dead individuals |
| 16 | (V_2_,S_HCW_) → (V_2_–1, S_HCW_ + 1) | $\left[ 1-\int_{t-\tau}^{t-\tau+\frac{1}{\phi}} \frac{\left( I\left( u \right)\beta_{I\to HCW}+{H\left( u \right)\beta}_{H\to HCW}+{D\left( u \right)\beta}_{D\to HCW} \right)}{N}\mathrm{du} \right]\xi_{1}S_{\mathrm{HCW}}\left( t-\tau\right)$ | Vaccinated healthcare workers reverting to the susceptible pool upon expiration of vaccine efficacy |
| 17 | (V_4_,S_G_) → (V_4_–1, S_G_ + 1) | $\left[ 1-\int_{t-\tau}^{t-\tau+\frac{1}{\phi}} \frac{\left( I\left( u \right)\beta_{I\to NHCW}+{H\left( u \right)\beta}_{H\to NHCW}+{D\left( u \right)\beta}_{D\to NHCW} \right)}{N}\mathrm{du} \right]\xi_{2}S_{G}\left( t-\tau\right)$ | Vaccinated individuals in the general population reverting to the susceptible pool upon expiration of vaccine efficacy |
