# Supplementary material for: Impact of prophylactic vaccination strategies on Ebola virus transmission: A modeling analysis
Source: PLoS One. 2020 Apr 27;15(4):e0230406. doi: 10.1371/journal.pone.0230406 (PMC7185698; doi:10.1371/journal.pone.0230406)
Supplement: S4 Table — (DOCX) [file pone.0230406.s006.docx]

**S4 Table. Description of parameters.**

| **Parameter** | **Description** |
| --- | --- |
| N | Size of the total population |
| S_HCW_ | HCW population |
| S_G_ | General population |
| E | Exposed population |
| I | Infected population |
| H | Hospitalized population |
| D | Dead |
| R | Recovered population |
| B | Dead and buried individuals |
| V_1_ | Vaccinated members of the HCW population who are yet to be protected |
| V_2_ | Vaccinated members of the HCW population who are protected |
| V_3_ | Vaccinated members of the general population who are yet to be protected |
| V_4_ | Vaccinated members of the general population who are protected |
| 1/σ | Mean latency period |
| 1/α | Mean duration from onset of infection to hospitalization |
| β_I→HCW_ | Transmission rate from infectious individuals to HCW (In days-1) |
| β_H→HCW_ | Transmission rate from hospitalized individuals to HCW (In days−1) |
| β_D→HCW_ | Transmission rate from dead but not buried individuals to HCW (In days−1) |
| β_I→NHCW_ | Transmission rate from infectious individuals to the non-HCW/general population (In days−1) |
| β_H→NHCW_ | Transmission rate from hospitalized individuals to the non-HCW/general population (In days−1) |
| β_D→NHCW_ | Transmission rate from dead but not buried individuals to the non-HCW/general population (In days−1) |
| δ_1_ | Case-fatality rate of non-hospitalized infectious individuals |
| δ_2_ | Case-fatality rate of hospitalized individuals |
| 1/γ | Mean duration from onset of infection to death/recovery |
| 1/γ_H_ | Mean duration from hospitalization to death/recovery |
| 1/γ_D_ | Mean duration from death to burial |
| τ | Durability of protection |
| 1/ϕ | Time to onset of protection |
| ξ_1_ | Rate of vaccination in the HCW population |
| ξ_2_ | Rate of vaccination in the general population |

Abbreviations: HCW, healthcare workers
