# Supplementary material for: Impact of prophylactic vaccination strategies on Ebola virus transmission: A modeling analysis
Source: PLoS One. 2020 Apr 27;15(4):e0230406. doi: 10.1371/journal.pone.0230406 (PMC7185698; doi:10.1371/journal.pone.0230406)
Supplement: S5 Table — (DOCX) [file pone.0230406.s007.docx]

**S5 Table. Sensitivity Analyses to Evaluate the Influence of Variability in Disease Parameters on the Impact of Vaccination of HCWs and the General Population.**

| **Model Parameter** | **0-95** | | | **95-186** | | | **186-587** | | |
| --- | --- | --- | --- | --- | --- | --- | --- | --- | --- |
|  | **Mean** | **Lower Bound** | **Upper Bound** | **Mean** | **Lower Bound** | **Upper Bound** | **Mean** | **Lower Bound** | **Upper Bound** |
| β_I→HCW_ | 117.8 | 106 | 130 | 15 | 13.5 | 16.5 | 5.1 | 4.59 | 5.61 |
| β_I→NHCW_ | 0.635 | 0.57 | 0.70 | 0.594 | 0.53 | 0.65 | 0.425 | 0.38 | 0.47 |
| β_H→HCW_ | 189.21 | 170 | 208 | 23.64 | 21.28 | 26 | 8.88 | 7.99 | 9.77 |
| β_H→NHCW_ | 0.0020 | 0.0018 | 0.0022 | 0.001 | 0.0009 | 0.0011 | 0.0005 | 0.0005 | 0.0006 |
| β_D→HCW_ | 0.0726 | 0.065 | 0.080 | 0.0511 | 0.046 | 0.056 | 0.045 | 0.041 | 0.05 |
| β_D→NHCW_ | 0.073 | 0.065 | 0.080 | 0.051 | 0.046 | 0.056 | 0.045 | 0.041 | 0.05 |
| δ_1_ | 0.46 | 0.42 | 0.51 | 0.21 | 0.19 | 0.23 | 0.68 | 0.61 | 0.75 |
| δ_2_ | 0.46 | 0.42 | 0.51 | 0.21 | 0.19 | 0.23 | 0.68 | 0.61 | 0.75 |
| Time to hospitalization (1/α) | 2.40 | 2.16 | 2.64 | 2.4 | 2.16 | 2.64 | 2.2 | 1.98 | 2.42 |
| Time from hospitalization to recovery/death (1/γ_H_) | 6.22 | 5.59 | 6.84 | 8.31 | 7.48 | 9.14 | 16 | 14.4 | 17.6 |
