# Supplementary figures and images for: Impact of prophylactic vaccination strategies on Ebola virus transmission: A modeling analysis
Source: PLoS One. 2020 Apr 27;15(4):e0230406. doi: 10.1371/journal.pone.0230406 (PMC7185698; doi:10.1371/journal.pone.0230406)

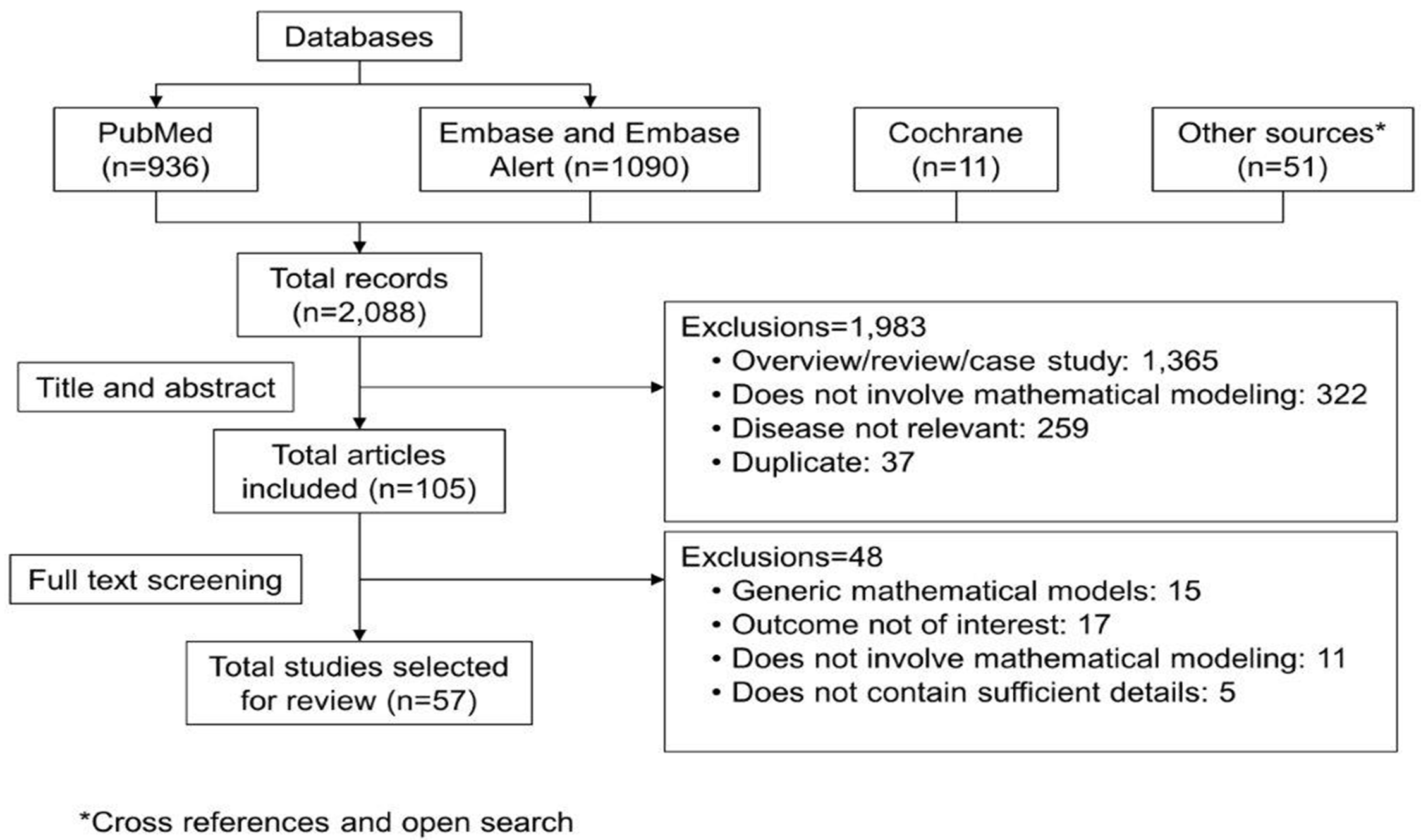

Supplement: S1 Fig — (TIF) [file pone.0230406.s008.tif]

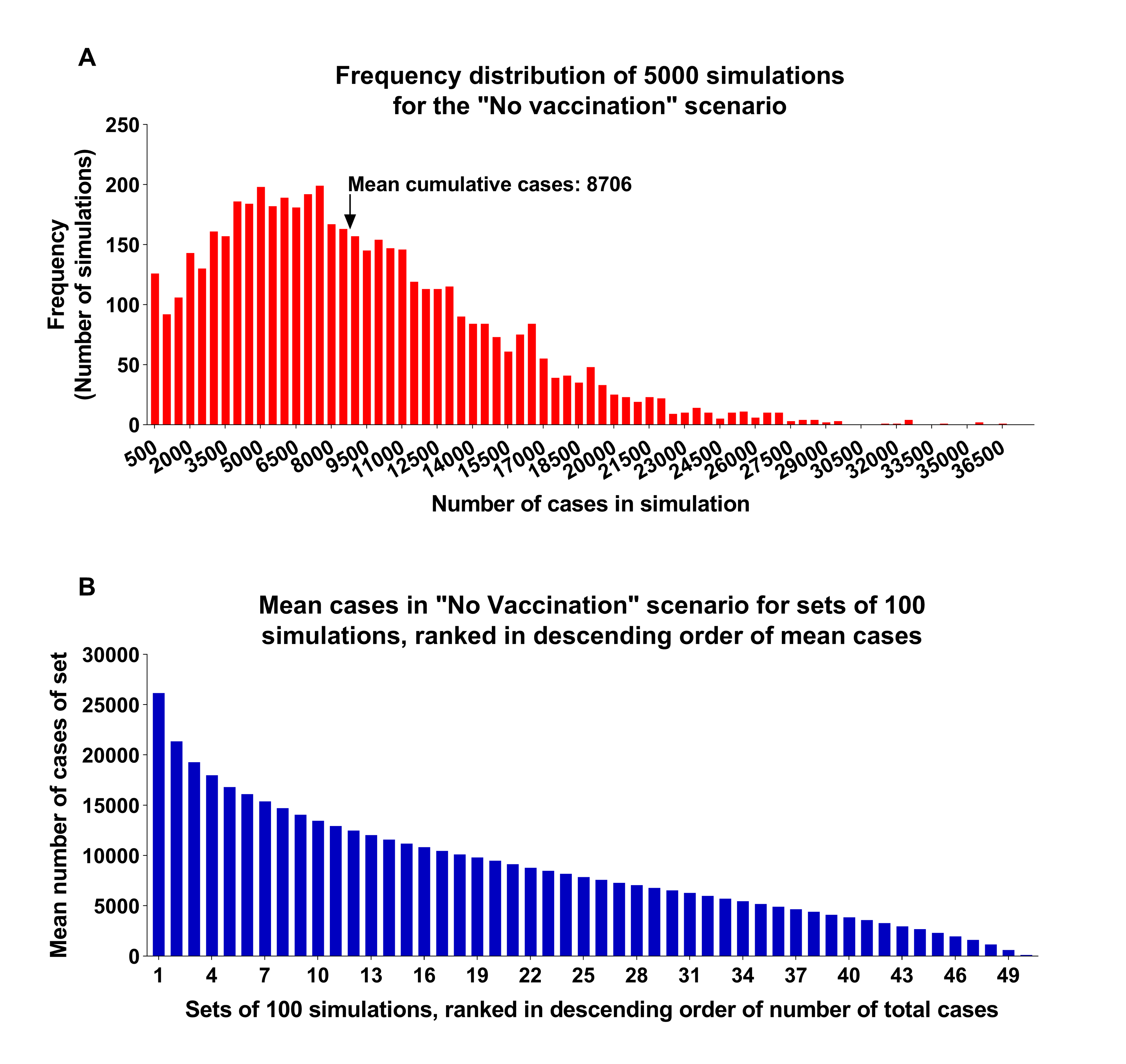

Supplement: S2 Fig — The data are based on 5000 simulations. (TIF) [file pone.0230406.s009.tif]

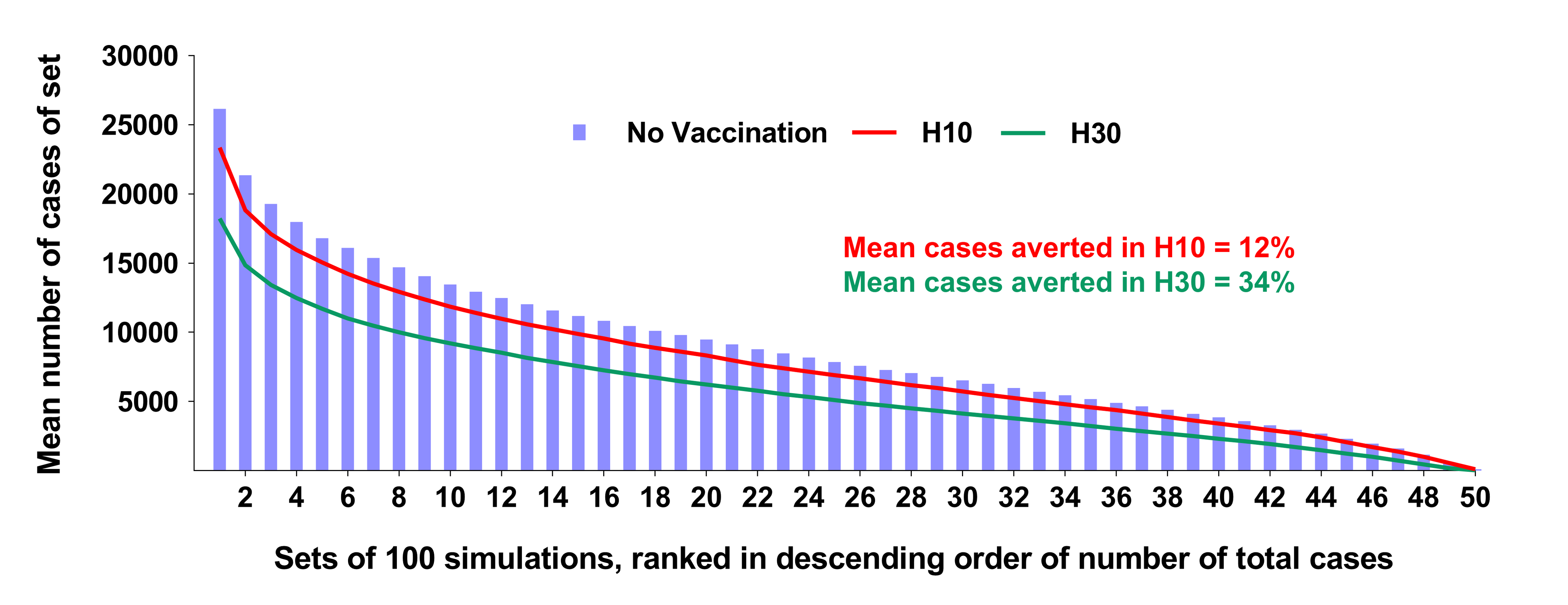

Supplement: S3 Fig — Results for scenarios in which 10% and 30% of HCW were vaccinated have been depicted. (TIF) [file pone.0230406.s010.tif]

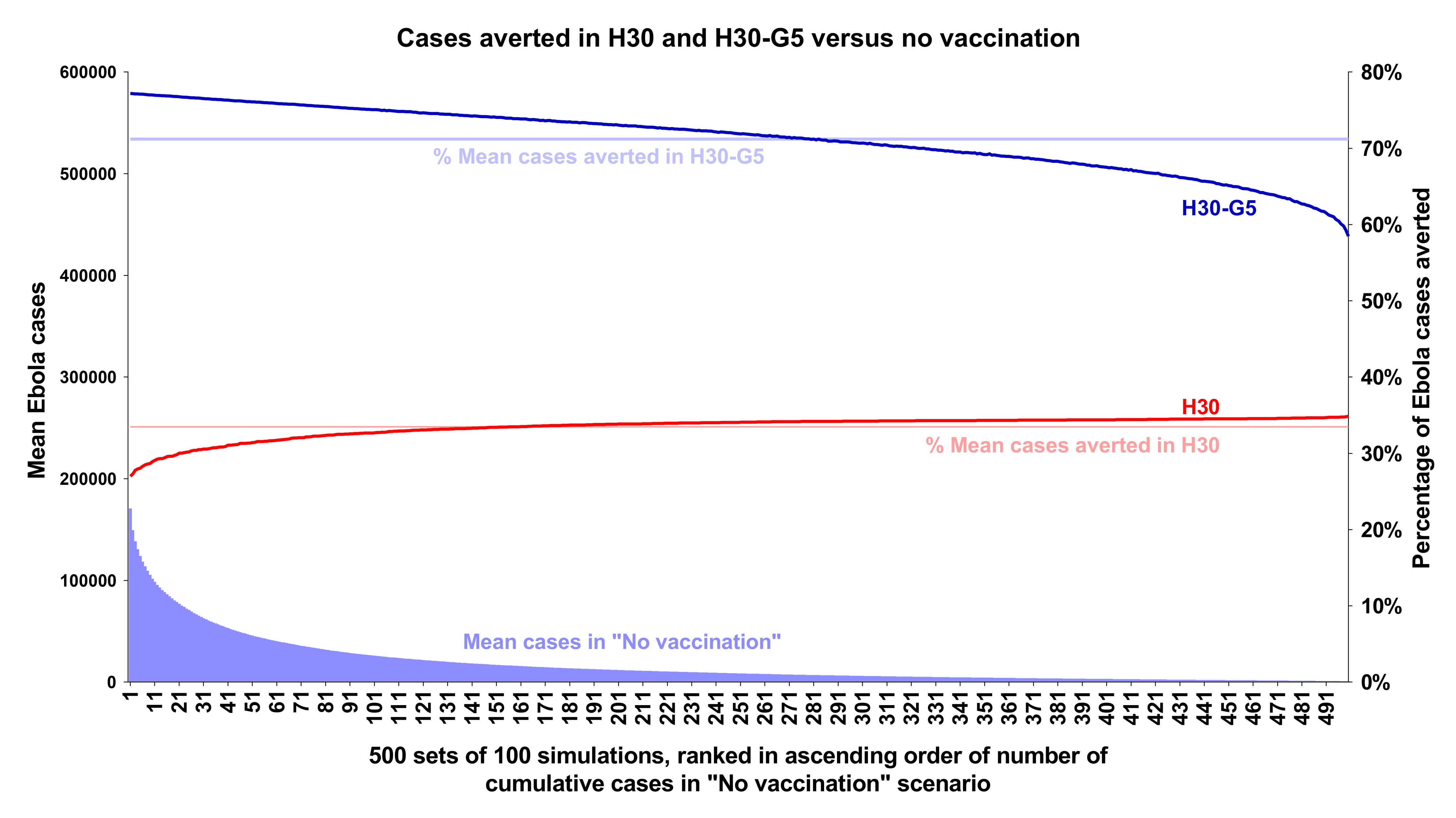

Supplement: S4 Fig — Monte Carlo simulation was carried out with random variations in these parameters being introduced (within a range of ±10%). (TIF) [file pone.0230406.s011.tif]

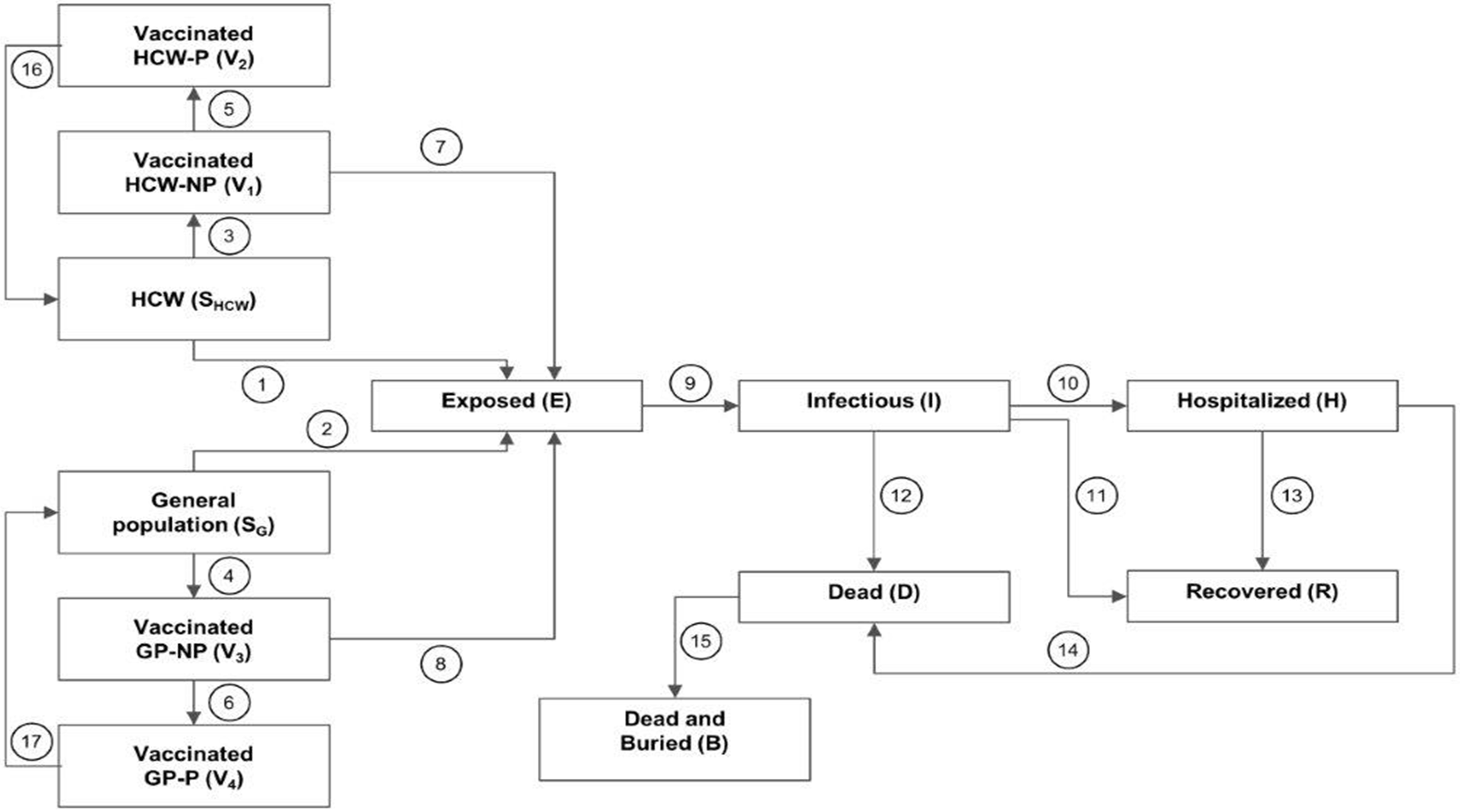

Supplement: S5 Fig — (TIF) [file pone.0230406.s012.tif]
